# Electronic and ring size effects of N-heterocyclic carbenes on the kinetics of ligand substitution reactions and DNA/protein interactions of their palladium(II) complexes

**DOI:** 10.1007/s10534-023-00507-8

**Published:** 2023-05-15

**Authors:** Reinner O. Omondi, Deogratius Jaganyi, Stephen O. Ojwach

**Affiliations:** 1 School of Chemistry and Physics, University of KwaZulu-Natal, Scottsville, Private Bag X01, Pietermaritzburg, 3209 South Africa; 2 School of Pure and Applied Sciences, Mount Kenya University, P.O. Box 342-01000, Thika, Kenya; 3 Department of Chemistry, Faculty of Applied Sciences, Durban University of Technology, P.O. Box 1334, Durban, 4000 South Africa

**Keywords:** Palladium(II) complexes, Substitution kinetics, DNA and BSA binding, Molecular docking

## Abstract

The synthesis, substitution kinetics and DNA/BSA interactions of four cationic Pd(II) complexes [Pd(**1**)Cl]BF_4_ (**Pd1**), [Pd(**2**)Cl]BF_4_ (**Pd2**), [Pd(**3**)Cl]BF_4_ (**Pd3**) and [Pd(**4**)Cl]BF_4_ (**Pd4**), derived from the reaction of [PdCl_2_(NCCH_3_)_2_] with ligands 2,6-bis(3-methylimidazolium-1-yl)pyridine dibromide (**1**), 2,6-bis(3-ethylimidazolium-1-yl)pyridine dibromide (**2**), 2,6-bis(1-methylimidazole-2-thione)pyridine (**3**), and 2,6-bis(1-ethylimidazole-2-thione)pyridine (**4**), respectively are reported. The complexes were characterised by various spectroscopic techniques and single crystal X-ray diffraction for compound **Pd2**. Kinetic reactivity of the complexes with the biologically relevant nucleophiles thiourea (**Tu**), L-methionine (**L-Met**) and guanosine 5′-monophosphate sodium salt (**5’-GMP**) was in the order: **Pd1** > **Pd2** > **Pd3** > **Pd4**, which was largely dependent on the electronic and ring size of the chelate ligands, consistent with Density functional theory (DFT) simulations. The interactions of the complexes with calf thymus DNA (CT-DNA) and bovine serum albumin (BSA) binding titrations showed strong binding. Both the experimental and in silico data reveal CT-DNA intercalative binding mode.

**Graphical abstract:**

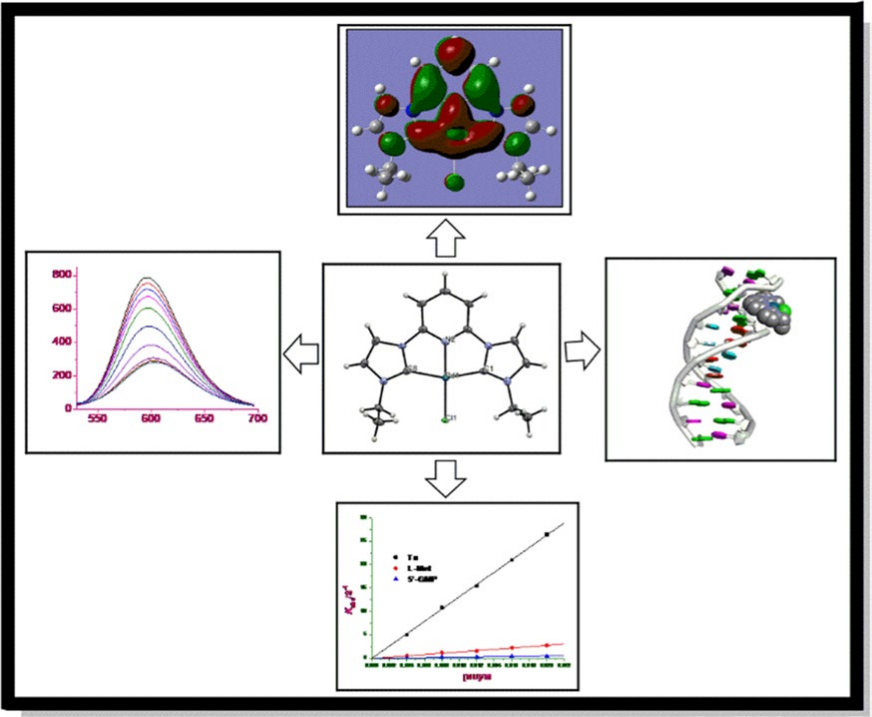

**Supplementary Information:**

The online version contains supplementary material available at 10.1007/s10534-023-00507-8.

## Introduction

N-heterocyclic carbenes (NHCs; CNC) and their derivatives (NHEs; ENE, where E = S, Se and Te) are versatile ligands used in the coordination chemistry of transition metals (Wang and Robinson 2011; Jia et al. 2009; Manjare et al. 2012; Collado-Martinez et al. 2016; Onar et al. 2019; Hussaini et al. 2019; Kumar et al. 2017; Ostrowska et al. 2021). The attractive features of their transition metal complexes have resulted to their wide range of applications in material science (Han et al. 2014; Zhang et al. 2015), catalysis (Ghavale et al. 2015; Jia et al. 2020) and therapeutics (Han et al. 2014; Hindi et al. 2009; Seliman et al. 2017; Jomaa et al. 2018). The NHCs and NHEs ligands are often used to stabilise organometallic centres, owing to their fascinating chemical properties such as strong σ-donating and poor (weak or even negligible) π-accepting abilities, saturation or aromaticity, forming complexes that are resistant to thermal decomposition (Manjare et al. 2012; Jia et al. 2015, 2020; Miecznikowski et al. 2012). The stronger σ-donor properties of NHEs in comparison to both NHC and phosphine is attributable to the presence of a larger contribution (66%) of the zwitterionic form (NHC^+^–E^−^) (Rani et al. 2017).

Metal-NHCs have demonstrated promising biological results (stemming from their favourable biocompatibility and molecular targeting properties), and among them Pd- NHC complexes have gain significant traction over the last decade (Bernd et al. 2020; Lee et al. 2015; Akkoç et al. 2017; Ghdhayeb et al. 2017). Pd-NHC compounds demonstrate appreciable stability under physiological conditions, which enhances their delivery to tumour tissues and thus minimising toxic side effects. Apart from their acceptable delivery method, Pd(II)-NHC complexes exhibit good solubility in the aqueous media (Al Nasr et al. 2020).

Che and the group (Fong et al. 2016) have reported the antitumour activity of a class of palladacyclic complexes supported by NHCs ligands. The results of the study revealed that the complexes are more active than palladacyclic derivatives with no NHCs ligands (i.e., with weaker σ-donors). The complexes also demonstrated high stability under physiological conditions. In addition, the authors found Pd(II)-NHCs to display better in vitro cytotoxicity than cisplatin in all the tested cancer lines. In a previous study, Gosh and co-workers (Ray et al. 2007), examined the antiproliferative properties of Pd(II)-NHC complexes. The compounds were found to have remarkable activity on MCF-7, HCT-116 and HeLa with better IC_50_ values than those of cisplatin. Noteworthy, most investigations have demonstrated that the main target for Pd-NHEs in cancer cells is DNA similar to cisplatin’s (Ray et al. 2007; Teyssot et al. 2009), with three distinct modes of interactions i.e., groove binding, intercalative, and electrostatic. These results encouraged us to consider Pd(II) complexes anchored on functionalised NHC and NHEs ligands. The substitution kinetics of the complexes with biological nucleophiles; thiourea (**Tu**), L-methionine (**L-Met**) and guanosine-5’-monophosphate (**5’-GMP**) are hereby reported. We also explored the interactions of the complexes with the DNA and BSA protein and their molecular docking studies.

## Experimental section

### Syntheses of palladium metal complexes

*[{2,6-bis(3-methylimidazolium-1-yl)pyridine dibromide}PdCl]BF*_*4*_* (****Pd1****)* To a solution of ligand 2,6-bis(3-methylimidazolium-1-yl)pyridine dibromide, **1** (0.50 g, 1.25 mmol) in CH_3_CN (30 mL) was added Ag_2_O (0.29 g, 1.25 mmol) and the reaction was stirred at 50 ºC for 24 h. To the mixture, AgBF_4_ (0.24 g, 1.26 mmol) was added, followed by PdCl_2_(MeCN)_2_ (0.32 g, 1.25 mmol) in a dark-room environment with stirring at 50 ºC for 24 h. After the reaction period, the mixture was cooled to room temperature and filtered. The filtrate was then evaporated in *vacuo* and the yellow residue washed with CH_2_Cl_2_ and diethyl ether to obtain compound **Pd1** as a brown-yellow solid. Yield: 0.42 g (72%). ^1^H NMR (400 MHz, DMSO-d_6_): δ_H_ (ppm): 3.91 (s, 6H, CH_3_); 7.61 (s, 2H, imid); 7.92 (d, ^3^J_HH_ = 8.2, 2H, py); 8.35 (s, 2H, imid); 8.51 (t, ^3^J_HH_ = 8.2, 1H, py). ^13^C NMR (DMSO-d_6_): δ_C_ (ppm): 36.51 (CH_3_); 108.69 (imidazole, CH); 117.93 (pyridine, CH); 125.34 (imidazole, CH); 146.47 (pyridine, CH); 150.25 (pyridine, CH); 166.35 (pyridine, C). FT-IR (cm^−1^): υ(C–H, aromatic) = 3615; υ(C–H, alkyl) = 3132; υ(C=N, imidazole) = 1619; υ(C=N, pyridine) = 1587; υ(C-N, imidazole) = 1033. LC MS/ESI^+^, *m/z *(%) = 382 (100% [M-BF4]^+^). Anal. Calcd (%) for C_13_H_15_BClF_4_N_5_Pd: C, 33.22; H, 3.22; N, 14.90%. Found: C, 33.51; H, 2.98; N, 14.74%.

*[{2,6-bis(3-ethylimidazolium-1-yl)pyridine dibromide}PdCl]BF*_*4*_* (****Pd2****)* Complex **Pd2** was synthesised in a similar fashion using ligand 2,6-bis(3-ethylimidazolium-1-yl)pyridine dibromide, **2** (0.53 g, 1.25 mmol), Ag_2_O (0.29 g, 1.25 mmol), AgBF_4_ (0.24 g, 1.26 mmol), and PdCl_2_(MeCN)_2_ (0.32 g, 1.25 mmol) to afford a brown-yellow solid. Further recrystallization in CH_2_Cl_2_/Et_2_O solution led to the formation of single-crystals suitable for X-ray crystallography analysis. Yield: 0.46 g (74%). ^1^H NMR (400 MHz, DMSO-d6): δ_H_ (ppm): 1.37 (t, ^3^J_HH_ = 7.2, 6H, CH_3_); 4.41 (dd, ^3^J_HH_ = 7.2, 4H, CH_2_); 7.75 (d, ^3^J_HH_ = 2.1, 2H, imidazole, CH); 7.94 (d, ^3^J_HH_ = 8.2, 2H, imidazole, CH); 8.38 (d, ^3^J_HH_ = 2.1, 2H, pyridine, CH); 8.52 (t, ^3^J_HH_ = 8.2, 1H, pyridine, CH). ^13^C NMR (DMSO-d_6_): δ_C_ (ppm): 16.14 (CH_3_); 44.43 (CH_2_); 108.73 (imidazole, CH); 118.22 (pyridine, CH); 123.67 (imidazole, CH); 146.46 (pyridine, CH); 150.34 (pyridine, CH); 165.88 (pyridine, C). FT-IR (cm^−1^): υ(C–H, aromatic) = 3138; υ(C–H, alkyl) = 2989; υ(C=N, imidazole) = 1619; υ(C=N, pyridine) = 1491; υ(C-N, imidazole) = 1035. LC MS/ESI^+^, *m/z *(%) = 410 (100% [M-BF4]^+^). Anal. Calcd (%) for C_15_H_19_BClF_4_N_5_Pd: C, 36.18, H, 3.85; N, 14.06. Found: C 35.81; H, 3.53, N, 14.34%.

*[{2,6-bis(3-methylimidazole-2-thione)pyridine}PdCl]BF*_*4*_* (****Pd3****)* This compound was prepared by introducing a mixture of ligand 2,6-bis(1-methylimidazole-2-thione)pyridine, **3** (0.12 g, 0.39 mmol) and NaBF_4_ (0.04 g, 0.39 mmol) in CH_2_Cl_2_ (10 mL) to a solution of [PdCl_2_(NCMe)]_2_ (0.10 g, 0.39 mmol) in CH_2_Cl_2_ (20 mL). The resultant mixture was then stirred at room temperature for 12 h and filtered to afford complex **Pd3** as an analytically pure compound. Yield: 0.14 g (67%). ^1^H NMR (400 MHz, DMSO-d_6_): δ_H_ (ppm): 3.80 (s, 6H, CH_3_); 7.85 (d, ^3^J_HH_ = 2.4, 2H, imidazole, CH); 8.10 (d, ^3^J_HH_ = 8.2, 2H, imidazole, CH); 8.21 (d, ^3^J_HH_ = 2.4, 2H, pyridine, CH); 8.67 (t, ^3^J_HH_ = 8.2, 1H, pyridine, CH). ^13^C NMR (DMSO-d_6_): δ_C_ (ppm): 35.71 (CH_3_), 116.80 (imidazole, CH), 117.53 (imidazole, CH), 120.12 (imidazole, CH), 140.17 (pyridine CH), 162.2 (pyridine, C), 170.53 (C = S). FT-IR (cm^−1^): υ(C–H, aromatic) = 3489; υ(C–H, alkyl) = 3102; υ(C=N, imidazole) = 1603 υ(C=N, pyridine) = 1473; υ(C = S, imidazole) = 1240, υ(C-N, imidazole) = 1019. LC MS/ESI^+^, *m/z *(%) = 489 (8% [(M-BF_4_) + 2Na]^+^). Anal. Calcd (%) for C_13_H_13_BClF_4_N_5_PdS_2_: C, 29.35; H, 2.46; N, 13.16; S, 12.05%. Found: C, 29.02; H, 2.17; 12.79, S, 12.26%.

*[{2,6-bis(3-ethylimidazole-2-thione)pyridine}PdCl]BF*_*4*_* (****Pd4****)* The procedure used in the preparation of **Pd3** was employed in the synthesis of **Pd4**, using ligand 2,6-bis(1-ethylimidazole-2-thione)pyridine,** 4** (0.13 g, 0.39 mmol), PdCl_2_(NCMe)_2_ (0.10 g, 0.39 mmol), and NaBF_4_ (0.04 g, 0.39 mmol). Yellow solid. Yield: 0.15 g (69%). ^1^H NMR (400 MHz, DMSO-d_6_): δ_H_ (ppm): 1.44 (t, ^3^J_HH_ = 7.3, 6H, CH_3_); 4.23 (dd, ^3^J_HH_ = 7.2, 4H, CH_2_); 7.97 (d, ^3^J_HH_ = 2.4, 2H, imidazole, CH); 8.13 (d, ^3^J_HH_ = 8.2, 2H, imidazole, CH); 8.25 (d, ^3^J_HH_ = 2.4, 2H, pyridine, CH); 8.69 (t, ^3^J_HH_ = 8.2, 1H, pyridine, CH). ^13^C NMR (DMSO-d_6_): δ_C_ (ppm): 13.80 (CH_3_), 44.54 (CH_2_), 116.72 (pyridine, CH), 117.55 (imidazole, CH), 120.81 (imidazole, CH), 140.12 (pyridine, CH), 164.66 (pyridine, C), 170.45 (C=S). FT-IR (cm^−1^): υ(C–H, aromatic) = 3546; υ(C–H, alkyl) = 3092; υ(C=N, imidazole) = 1600; υ(C=N, pyridine) = 1458; υ(C = S) = 1153; υ(C-N, imidazole) = 1021. LC MS/ESI^+^, *m/z *(%) = 474 (55% [M-BF4]^+^). Anal. Calcd (%) for C_15_H_17_BClF_4_N_5_PdS_2_: C, 32.16; H, 3.06; N, 12.50; S, 11.45%. Found: C, 32.44; H, 3.23; N, 12.15; S, 11.76%.

## Results and discussion

### Syntheses and structural characterisation of Pd(II) complexes

**Scheme 1:**
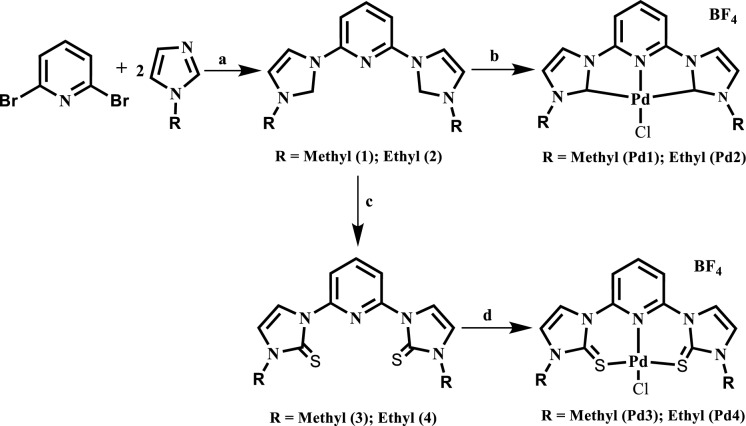
Synthetic pathways map for the ligands and their respective Pd(II) complexes. *Reagents and conditions*; **a** neat conditions, 150 °C, 20 h; **b** Ag_2_O, PdCl_2_(NCMe)_2_, AgBF_4_, MeCN, 50 °C; **c** S_8_ powder, K_2_CO_3_, MeOH, 8 h; **d** PdCl_2_(NCCH_3_)_2_, NaBF_4_, CH_2_Cl_2_, room temperature, 12 h

The preparation of ligands **1** and** 2** was carried out by employing the previously described method (Chen and Lin 2000), through the reaction of 2,6-dibromopyridine with the corresponding imidazoles under solvent free conditions at 150 °C (Scheme 1). In contrast, ligands **3** and **4** were prepared according to the well-established synthetic procedure (Jia et al. 2015), via the condensation reactions of pyridine bridged imidazolium dibromide derivatives with sulfur powder in the presence of K_2_CO_3_ (Scheme 1) and were isolated in moderate yields. Detailed synthetic protocols, their spectroscopic and analytical data are given in the supplementary section. The dicarbene Pd(II) complexes **Pd1** and **Pd2**, were obtained in the sequential one-pot reaction of PdCl_2_(NCMe)_2_, Ag_2_O, AgBF_4_ with the respective ligands **1** and **2** in MeCN at 50 °C as described in Scheme 1. On the contrary, the reactions of **3** and **4** with equimolar amounts of [PdCl_2_(NCMe)_2_] in the presence of NaBF_4_ in CH_2_Cl_2_ produced the corresponding complexes **Pd3** and **Pd4** (Scheme 1).

The formation and identities of the complexes were confirmed by ^1^H, ^13^C NMR (Figs. S1-S16) and FT-IR (**Figs. S17–S25**) spectroscopies, mass spectrometry (Figs. S26–S32), elemental analyses, and single crystal X-ray analyses (for **Pd2**). For instance, the ^1^H NMR spectrum of free ligand **1** displayed a singlet peak at* δ* 10.63 ppm, ascribed to the imidazolium CH proton and upon complexation to form complex **Pd1**, the peak disappeared (Fig. S9), indicative of the coordination of the CH moiety to the Pd(II) atom. Likewise, from ^13^C NMR spectra, the downfield shifts of the carbene carbon at 145.20 ppm (**1**) to 166.35 pm (**Pd1**) is consistent with the formation of the complex (Fig. S16). Similarly, the successful coordination of the ligands to produce the corresponding Pd(II) complexes was determined from their respective FT-IR spectra. For instance, a shift of the absorption bands at 1605 cm^−1^ (C=N, imidazole) and 1531 cm^−1^ (C=N, pyridine) in **1** to higher frequency values of 1619 and 1587 cm^−1^ in **Pd1** respectively (Fig. S25) affirmed the coordination of the Pd(II) ion to the nitrogen atom of pyridine and the carbene in the imidazolium unit. Positive electron ionisation mass spectrometry (ESI-LC–MS) further proved the formation of the Pd-complexes. For example, the LC–MS spectrum for **Pd1** gave characteristic peak at *m/z* at 382 (**Fig**. **S29a**) corresponding to its exact mass of 382.01. In addition, the experimental isotopic mass distribution patterns were in congruous with the calculated isotopic mass distributions (**Fig**. **S29b**). Elemental analyses data for all the complexes showed close agreement with the theoretical calculations validating the proposed structures and their purity.

### X-ray diffraction analysis

**Fig. 1:**
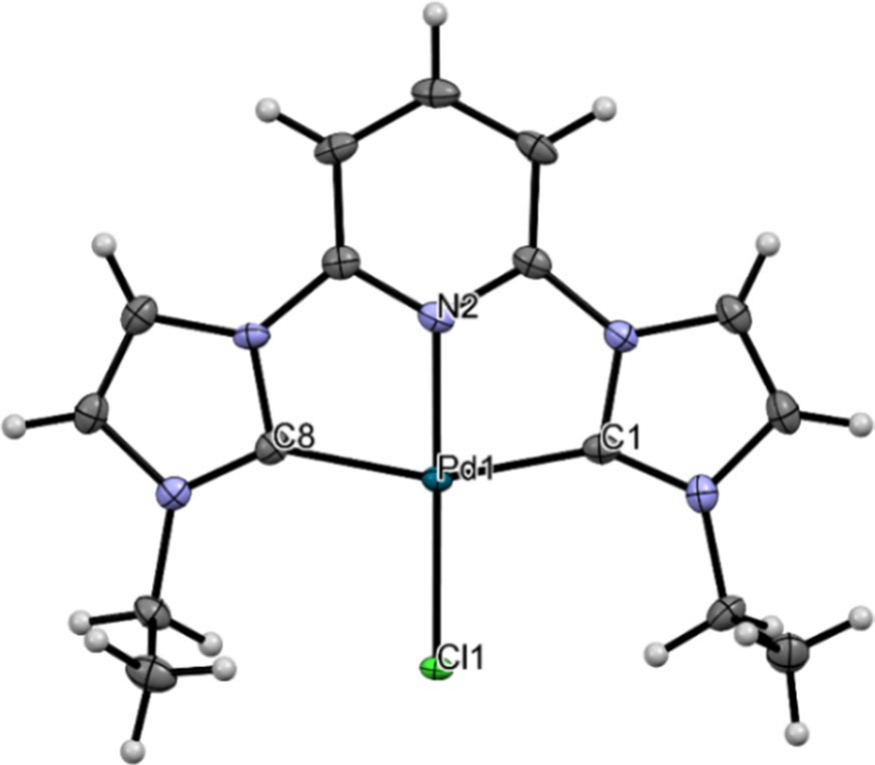
Molecular structure of **Pd2**, depicting the coordination environment of Pd(II) centre. The hydrogen atoms are shown as spheres of common arbitrary radius. Selected bond lengths [Å]: Pd(1)–N(2), 1.966(3); Pd(1)–C(1), 2.032(3); Pd(1)–C(8), 2.035(3); Pd(1)–Cl(1), 2.3033(8). Selected bond angles [°]: N(2)–Pd(1)-C(1), 79.46(12); N(2)–Pd(1)–C(8), 79.03(13); C(1)–Pd(1)–C(8), 158.50(14); N(2)–Pd(1)–Cl(1), 179.15(9); C(1)–Pd(1)–Cl(1), 100.91(10); C(8)–Pd(1)–Cl(1), 100.59(10)

Single crystals suitable for crystallographic analyses of complex **Pd2** were obtained by slow diffusion of diethyl ether/acetonitrile layered solutions. The molecular structure of complex **Pd2** is shown in Fig. 1, while the crystallographic data and structural refinement parameters are summarised **in Table S1.**

Complex **Pd2** crystallised in the monoclinic space group, P 21/n. The molecular structure reveals one tridentate ligand unit and Cl atom to give a nominally square planar geometry. The dihedral angles N(2)–Pd(1)–C(1) of 79.46(12)° and N(2)–Pd(1)–C(8) of 79.03(13)° deviate considerably from the expected 90° for a perfect square planar geometry. Similarly, the bite angles C(1)–Pd(1)–C(8) of 158.50(14)° depart significantly from 180°, consistent with a distorted square planar geometry. Therefore, the geometry around the Pd(II) ion could be described as *pseudo* square planar (Yang et al. 2007). The average bond distance Pd–C_av_ (2.033 Å) is within the range 1.996 ± 0.038 Å averaged for 15 related Pd structures (Groom et al. 2016). The bond length Pd(1)–Cl(1) 2.303(8) Å compares reasonably well with the mean distance of 2.365 ± 0.022 Å calculated from 14 related structures (Cooper 2020).

### DFT calculations

For an in depth understanding of the structural properties of the complexes, quantum chemical calculations were performed. The modelled geometry structures, frontier orbital density distributions (HOMOs and LUMOs), and planarity of the complexes are given in Fig. S33, while a summary of the selected geometrical data are provided in Table S2. Both the HOMO and LUMO orbitals of complexes **Pd1** and **Pd2** reveal that the electron density is centred primarily on the Pd(II) metal ions, chelating ligands, and partially on the chlorido ligands. On the other hand, the HOMOs and LUMOs of compounds **Pd3** and **Pd4** are significantly concentrated on the metal centres, imidazolyl units and the chlorido atoms. Notably, the significant distribution of the LUMOs on the Pd-metal centre, indicate the potential σ-donor ability of the chelating ligand. The formation of a more electron rich Pd(II) metal centre stabilises the ground state, while destabilising the five-coordinate transition state. Moreover, the chemical reactivity of the complexes is related to the ΔE_LUMO–HOMO_ energy gap, where compounds with smaller gaps were more reactive than those with large gaps. The optimized geometries of the complexes reveal that **Pd3** and **Pd4** suffer distortion to accommodate steric repulsions, causing the twisting of the chelating ligands and hence loss of planarity.

### Solution stability studies

The stability of complexes **Pd1–Pd4** under *pseudo* physiological conditions (50 μM Tris buffer containing 50 mM NaCl, pH 7.2) were assessed by time-dependent UV–Vis absorption spectroscopy. It has been shown that the interactions of the metal-complexes with water molecules influence their rates of substitution kinetics and resultant bio-activity (Zhao et al. 2017). As observed in Fig. S34, the UV–Vis spectra of the complexes showed no significant changes in MLCT absorption bands (both in intensity and position), ascribable to their non-interaction with H_2_O molecules. Thus, the compounds displayed sufficient stability in the aqueous solution for at least 72 h.

### Electrochemical studies

Redox properties of the complexes were studied by cyclic voltammetry (CV) under N_2_, with potential range of – 2.0 to 2.0 V. Scan rate dependency of the CVs of the complexes are indicated in **Fig. S35**. The cyclic voltammograms of the complexes are suggestive of irreversible reductive processes (consisting of two electron transfer) occurring at –1.2, – 1.4, –0.9, and –1.0 V for **Pd1**–**Pd4**, respectively. The irreversible cathodic waves of the complexes were associated with the reduction of Pd(II)/(0), assigned to the sigma-donor nature of ligand scaffold, towards the metal centre (Shabbir et al. 2016). This is also supported by the relatively higher HOMOs energy for the complexes with ethyl substituents (**Pd2** and **Pd4**) than those methyl groups (**Pd1** and **Pd3**).

### Kinetic and mechanistic study

#### Concentration effect

**Fig. 2:**
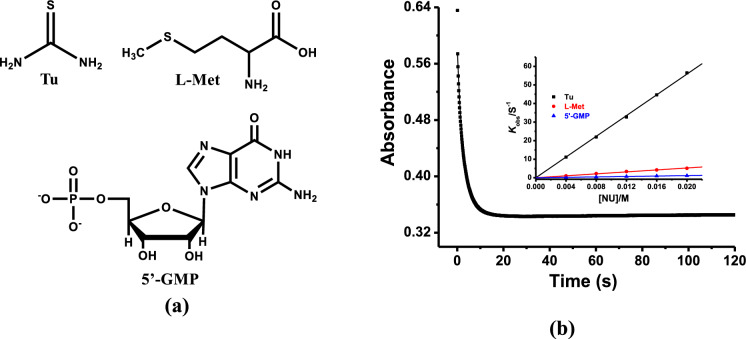
**a** Chemical structures for the entering ligands. **b** Time resolved stopped-flow kinetic trace at 345 nm of **Pd1**, at T = 298 K, 50 μM Tris–HCl buffer (pH = 7.2), and 50 mM NaCl {Inset: plot of *k*_obs_ vs [Nu]}

The reactivity of the complexes towards biological nucleophiles (i.e., **Tu**, **L-Met**, and **5’-GMP, **Fig. 2) were evaluated spectrophotometrically at physiological conditions. Typical kinetic trace recorded from stopped-flow spectrophotometer for the reaction of **Pd1** with **Tu** is shown in Fig. 2. All the kinetic profiles fitted well on a single-exponential decay function to generate the observed *pseudo*-first-order rate constants (*k*_*obs*_) using equation (S1), signifying that the reactions were first-order. The *k*_obs_ values obtained were plotted against nucleophiles concentration [Nu]. Representative plots of *k*_obs_ against [Nu] obtained for **Pd1** at 298 K is presented in Fig. 2b, while the spectra of complexes **Pd2**-**Pd4** are displayed in Fig. S36. A linear dependence of *k*_obs_ on [Nu] with zero intercept was observed in all the plots, indicative of irreversible or non-solvotic pathways (Bellam et al. 2019). The second-order rate constants (*k*_*2*_) were obtained from the dependence of *k*_*obs*_ on [Nu] using equation (S2), and the values are provided in Table 1. The relationship between *k*_*obs*_ and [Nu] can best be illustrated by equation (S3).

**Table 1:** Second order rate constants, *k*_2_, and activation parameters for the substitution reactions of **Pd1**–**Pd4** with **Tu**, **L-met** and **5’-GMP** at pH = 7.2 (50 μM Tris–HCl buffer and 50 mM NaCl)

Complex	Nu	*k*_2_/M^−1^ s^−1^	Δ*H*^≠^/ kJ mol^−1^	− Δ*S*^*≠*^/Jmol^−1^ K^−1^	ΔG^≠^_25 °C_/kJ mol^−1^
**Pd1**	**Tu**	2791 ± 19	14 ± 1	132 ± 4	53 ± 3
	**L-Met**	263 ± 5	18 ± 1	137 ± 3	59 ± 2
	**5’-GMP**	58 ± 1	26 ± 1	124 ± 4	63 ± 3
**Pd2**	**Tu**	2122 ± 13	15 ± 1	131 ± 3	54 ± 2
	**L-Met**	204 ± 4	21 ± 1	132 ± 4	60 ± 3
	**5’-GMP**	44 ± 2	26 ± 1	125 ± 3	63 ± 2
**Pd3**	**Tu**	1315 ± 9	28 ± 1	90 ± 3	55 ± 2
	**L-Met**	139 ± 3	41 ± 1	66 ± 3	61 ± 2
	**5’-GMP**	27 ± 2	44 ± 1	70 ± 3	65 ± 2
**Pd4**	**Tu**	930 ± 7	29 ± 2	89 ± 5	56 ± 4
	**L-Met**	108 ± 4	40 ± 1	71 ± 3	61 ± 2
	**5’-GMP**	22 ± 2	44 ± 1	71 ± 3	65 ± 2

From the second-order rate constants values, *k*_2_, provided in Table 1, the reactivity of the Pd(II) complexes increases marginally by factors ranging from 1.3 to 1.6. The trend of the reactivity of the complexes decreases following the order; **Pd1** > **Pd2** > **Pd3** > **Pd4**. The observed reactivity trend can be ascribed to both electronic and steric contributions of the spectator ligand(s). Notably, the lower reactivities of complexes **Pd2** & **Pd4** in relation to complexes **Pd1** and **Pd3** respectively is attributed to the superior σ-donation capability of the ethyl substituents (**Pd2** and **Pd4**) than the methyl groups in **Pd1** and **Pd3**. The net effect is the accumulation of electron density on the Pd metal centre, which hinders the incoming nucleophiles through electron–electron repulsions. The reasoning is consistent with the raised E_HOMO_ of **Pd2** in comparison to **Pd1** (Table S2). This is also reflected by the positive NBO charges of Pd(II) ions which decrease from 0.276 (**Pd1**) to 0.262 (**Pd2**) as shown in **Table S2.**

The argument is well validated by the raised E_HOMO_ values which are ordered as; **Pd3** (− 6.6734 eV) < **Pd4** (− 6.6007 eV), Table 2. The observed higher reactivity of C^N^C (**Pd1** and **Pd2)** in comparison to the S^N^S analogues **Pd3** and **Pd4** could also be due to the rigid five-membered ring and planarity in complexes **Pd1** and **Pd2** (**Fig. S33**). The smaller dihedral angles for N-Pd–C of 79.252° and 79.281° for **Pd1** and **Pd2** respectively (**Table S2**), result in strained chelate framework, consistent with enhanced intrinsic reactivities. In contrast, the structures of **Pd3** and **Pd4** are twisted out of the mean plane (**Fig**. **S33**), at dihedral angles, for instance N-Pd-S of 88.812 ° (**Pd3**) and 88.565 ° (**Pd4**) (**Table S2**). The boat-shaped conformation of **Pd3** and **Pd4** introduces steric effects and thereby impeding nucleophilic attack. In addition, the analysis of the of NBO charges on the Pd(II) depicts fairly unusual negative values of − 0.053 for **Pd3** and − 0.061 for **Pd4**, tenably due to the fact that S-atom is more polarisable than C-atom. Notably, the S-atom is larger and have more loosely held electrons than the C-atom, and thus is more willing to share the electron density with the Pd(II) ion through the σ-framework (Matta, Gillespie 2002).

**Table 2:** DNA binding constants and number of binding sites for Pd(II) complexes

Complex	UV–Vis titration		EB fluorescence quenching titration				
	*K*_b_ (10^5^ M^−1^)	ΔG/k J mol^−1^	*K*_sv_ (10^4^ M^−1^)	*K*_app_ (10^9^ M^−1^)	*k*_q_ (10^12^ M^−1^ s^−1^)	*K*_F_ (10^5^ M^−1^)	*n*
**Pd1**	31.24 ± 0.81	−37.05	7.79 ± 0.55	2.00 ± 0.23	3.39 ± 0.19	2.26 ± 0.17	1.13
**Pd2**	2.30 ± 0.11	− 30.59	6.70 ± 0.39	1.68 ± 0.26	2.91 ± 0.19	1.20 ± 0.14	1.10
**Pd3**	2.12 ± 0.13	− 30.39	4.80 ± 0.20	1.56 ± 0.08	2.08 ± 0.21	0.89 ± 0.06	0.79
**Pd4**	1.54 ± 0.13	− 29.60	0.63 ± 0.04	0.30 ± 0.05	0.28 ± 0.01	0.10 ± 0.03	0.81

The trend in the nucleophilicity of the entering ligands decreases in the form **Tu > L-Met > 5´-GMP** (Table 1), highlighting that the reactivity is controlled by both electronic and steric effects. Sulfur entering ligands (**Tu** and **L-Met**) show higher lability than the nitrogen entering ligand (**5’-GMP**). The observation is attributable to the soft-acid nature of Pd(II), which has a higher attraction for sulfur compounds (soft bases) (Jovanović et al. 2016). The entering ligand, **Tu** exhibit the highest reactivity because it combines the properties of the thioethers (function as σ-donors and π-acceptors), and thiolates (acts as π-donors) (Murray and Hartley 1981). In addition, the two amine groups in **Tu** in relation to **L-Met** improves the nucleophilicity on the S-atom as opposed to the methyl substituent. The bulkiness of **5′-GMP** hinders it from competing with **Tu** (least sterically demanding) and **L-Met** (moderate steric demands).

#### Temperature effect and iso-kinetic relationship

**Fig. 3:**
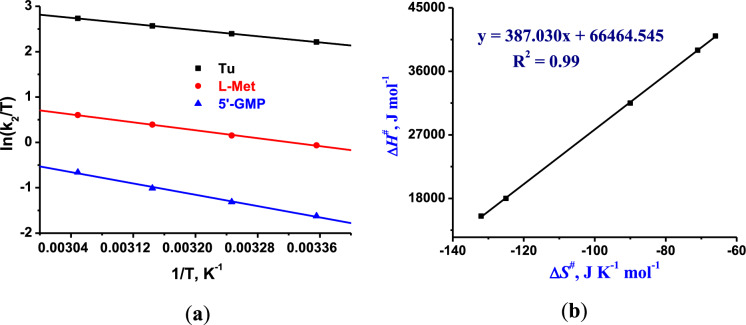
**A)** Plots for the reaction of **Pd1** with the nucleophiles at pH = 7.2 (50 μM Tris–HCl buffer and 50 mM NaCl) and temperature range of 298–318 K. **b** *Iso*-kinetic plots for **Pd1**-**Pd4** with the biological nucleophiles

To ascertain the mechanism of the substitution process followed by the complexes, the reaction temperatures were varied from 298 to 318 K. The enthalpy of activation (ΔH^≠^), entropy of activation (∆*S*^≠^) were computed from equation (S4). Gibbs free energy of activation (ΔG^≠^_25 °C_) was derived from equation (S5). Representative plots obtained for **Pd1** are given in Fig. 3a (plots for **Pd2**-**Pd4** are shown in Fig. S37). The calculated values of thermal parameters are depicted in Table 1. The higher reactivity of **Pd1** and **Pd2** in relation to **Pd3** and **Pd4** is attributed to the small values of Δ*H*^≠^ (Table 1), signifying low energy barrier that is associated with the process of the formation of bonds in the transition state. The small positive values Δ*H*^≠^ and the large negative values ∆*S*^#^ (Table 1) indicate an associative mechanism, where Pd-Cl bond breakage and Pd-nucleophile bonds formation are concerted (Atwood 1997). The large sensitivity of the second order rate constants to the nucleophiles is consistent with an associative mode of ligand substitution reaction (Petrović et al. 2012). The magnitude of ΔG^≠^_25 °C_ (obtained from Eq. 5) for the reactions of complexes **Pd1**–**Pd4** with the three biological nucleophiles are all comparable, indicating that the reactions follow a similar associative mechanism (Bellam et al. 2019, 2018).

Linear plots of Δ*H*^≠^ and ∆*S*^#^ for the kinetic reactions displayed the existence of a linear free energy relationship (LFER) between the two thermal parameters (Fig. 3b). The *iso*-kinetic temperatures and Gibbs free energies (ΔG^≠^) were respectively determined from the slopes and intercepts of the plots. The *iso*-kinetic temperature and ΔG^≠^ were predicted at 387.03 K, and at 66,464.54 kJ mol^−1^, respectively. Similarly, LFER/isokinetic plots illustrate that the kinetic reactivity of the complexes followed the same associative mechanism (Bellam et al. 2019, 2018).

### CT-DNA interactions studies

#### UV–Vis absorption spectral studies

**Fig. 4:**
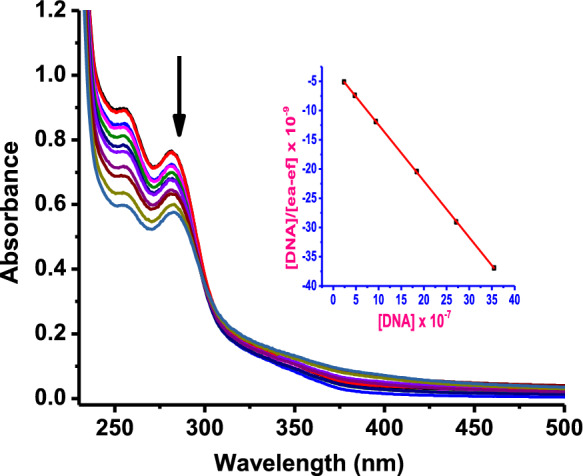
Absorption spectral changes of **Pd1** [20 µM] in Tris buffer solution at pH = 7.2, with the successive addition of CT-DNA [0–80 μM], at room temperature. The arrow indicates absorbance changes with the increasing CT-DNA concentration. Inset: plot of [CT-DNA] versus [DNA]/(ε_a_ – ε_f_)

Electronic absorption spectroscopy studies were performed to determine the mode and extent of binding of the complexes to CT-DNA. Typical absorption spectral titration curves of complex **Pd1** in the absence and presence of DNA, at varying concentration are shown in Fig. 4, while the spectra for compounds **Pd2**–**Pd4** are given in **Fig. S38**. The spectra of these complexes displayed well-resolved bands in the range 250–300 nm, arising from the intra-ligand π → π* charge transfer transitions (Sathyadevi et al. 2011).

Upon the addition of CT-DNA, the bands of complexes **Pd1**–**Pd4** demonstrated significant reduction in the absorption bands (hypochromism of ∼ 5–35%), demonstrating the existence of intercalative mode of binding (Balakrishnan et al. 2019). Of note such spectral changes are usually observed upon binding to guanine N_7_ atom (Protas et al. 2018). The extent and strength of intrinsic binding constants (*K*_*b*_) of the complexes to CT-DNA were determined from Wolfe-Shimer equation (S6) and the results are provided in Table 2. The *K*_b_ values of (31.24 ± 0.81) × 10^5^ (**Pd1**), (2.30 ± 0.11) × 10^5^ (**Pd2**), (2.12 ± 0.13) × 10^5^ (**Pd3**), and (1.54 ± 0.13) × 10^5^ M^−1^ (**Pd4**), compare well with related Pd(II) complexes obtained in literature (Franich et al. 2019; Karami et al. 2019; Mitra et al. 2018). Noteworthy, intercalative binding mode is largely sensitive to planarity of the chelating ligand(s) available for stacking, thus explaining the lower binding affinity for **Pd3** and **Pd4** (which suffer from distortion due to ring strain as depicted in the DFT Fig. S33). The free energy (∆G) of the complex-DNA adduct was evaluated using van't Hoff equation (S7), and the results are presented in Table 2. The calculated negative ∆G values of **Pd1**–**Pd4** highlight the energetically favourable and spontaneity of the Pd(II) complexes towards CT-DNA (Mukherjee et al. 2017; Karami et al. 2018).

#### Competitive fluorescence measurements for CT-DNA

**Fig. 5:**
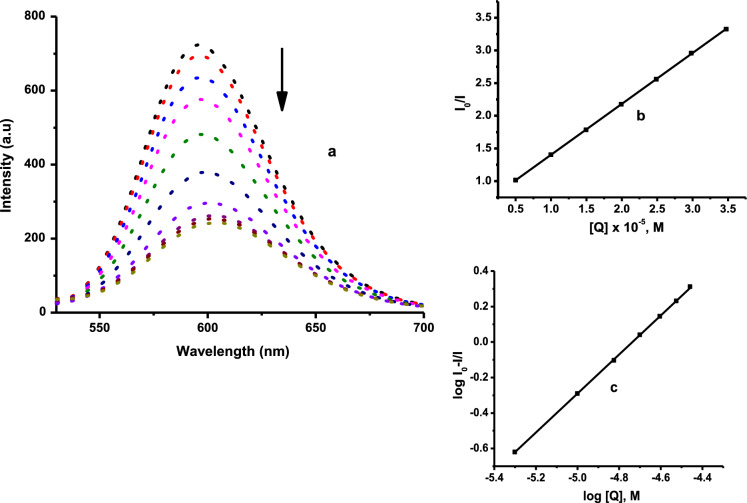
**a** The effects of addition of **Pd1** on the emission intensity of EB bound to CT-DNA at varying concentrations. [EB] = 20 μM, [CTDNA] = 20 μM and [**Pd1**] = 0–200 μM. The arrow shows the changes on addition of metal complex. **b** Stern–Volmer plot of *I*_o_/*I* versus [Q]. **(c)** Scatchard plot of log[(*I*_o_–I)/*I*] versus log[Q]

With the aim to further confirm the mode of binding between CT-DNA and complexes **Pd1**–**Pd4**, fluorescent-quenching assay based on the EB displacement from EB-DNA adduct was performed. Changes in the fluorescence spectra of EB-DNA composite in the absence and presence of varying concentration of the complexes is indicated in Fig. 5 for **Pd1** and Figs. S39–S41 for **Pd2**–**Pd4**, respectively. The incremental additions of complexes **Pd1**–**Pd4** to EB-CT-DNA system caused appreciable decrease in the fluorescence intensity (35–45%), suggestive of the complexes binding abilities to the DNA (Tarushi et al. 2013; Koumousi et al. 2012). The Stern–Volmer quenching constant (*K*_sv_) and bimolecular quenching rate constant (*k*_q_), were calculated from the Stern–Volmer equation (S8), and the data are given in Table 2. The *K*_sv_ values of **Pd1**–**Pd4** (magnitude 10^3^–10^4^ M^−1^), signifying that the studied complexes can displace bound EB from the CT-DNA via intercalative mode (Table 2) (Wei et al. 2020). The values of apparent binding constant (*K*_*app*_) obtained from equation (S9) are presented in Table 2. The calculated *K*_app_ values (magnitude 10^8^- 10^9^ M^−1^) are higher than the values for classical intercalators binding constant (10^7^ M^−1^) (Cory et al. 1985), confirming a strong intercalative interaction mode. The *K*_*q*_ values for the Pd-complexes found in the magnitude (10^11^–10^12^ M^−1^ s^−1^), are greater than most known dynamic fluorescent biopolymers (10^10^ M^−1^ s^−1^), indicating a static quenching mechanism (Mitra et al. 2018). The DNA binding constant, *K*_F_ and the number of binding sites per nucleotide, *n* were derived from the Scatchard equation (S10). The calculated *K*_*F*_ values of 1.00–22.60 × 10^4^ M^−1^, signify strong intercalative mode of binding. The n values which are near 1, show the presence of a single independent binding site in the CT-DNA for all the studied Pd(II) complexes. The results of the competitive fluorescence measurements are in good accord with the data obtained from the UV–Vis studies, showing that the complexes considerably interact with DNA in intercalative manner. The perturbation of the complexes on the base-stacking of CT-DNA follows the order; **Pd1** > **Pd2** > **Pd3** > **Pd4**, consistent with the substitution kinetics trend (Table 3).

**Table 3:** BSA binding constants and number of binding sites for the Pd(II) complexes

Complex	K_sv_ (10^5^ M^−1^)	k_q_ (10^13^ M^−1^ s^−1^)	K_F_ (10^3^ M^−1^)	n
Pd1	25.53 ± 0.92	11.10 ± 0.45	10.01 ± 0.38	0.77
Pd2	16.11 ± 0.90	7.01 ± 0.36	5.86 ± 0.32	0.78
Pd3	9.08 ± 0.31	3.95 ± 0.28	3.85 ± 0.30	0.84
Pd4	8.78 ± 0.33	3.82 ± 0.26	1.10 ± 0.21	0.68

### BSA interactions

#### Fluorescence quenching measurements

**Fig. 6:**
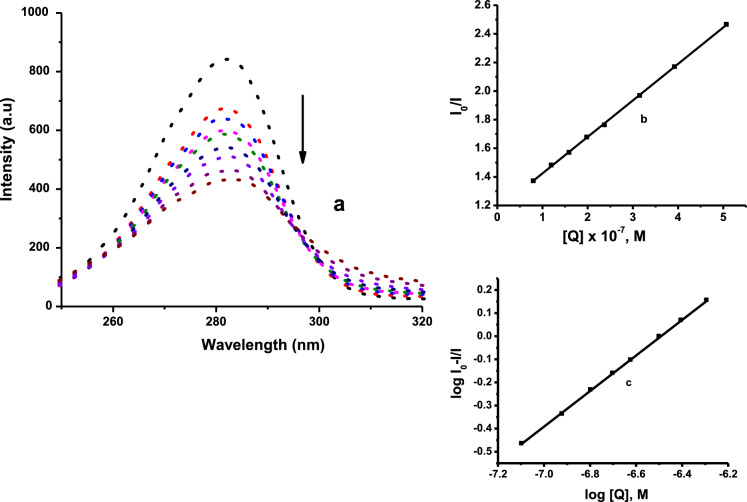
**a** Fluorescence emission spectrum of BSA in the absence and presence of **Pd1**: [BSA] = 1.2 μM and [**Pd1**] = 0–200 μM. The arrow shows the changes on addition of varying quantities of **Pd1**. **b** Stern–Volmer plot of *I*_o_/*I* versus [Q]. **c** Scatchard plot of log[(*I*_o_–I)/*I*] versus log[Q]

Serum albumin is the most essential circulating protein in the blood plasma, among other functions it is responsible for the distribution of drugs to their pharmacological target (Elsayed et al. 2022). The fluorescence emission spectrum for **Pd1** is shown in Fig. 6, while the spectra for **Pd2**–**Pd4** are provided in Figs. S42–S44, respectively. The intensity of the characteristic broad emission band at 281 nm shows notable decreasing trends with increasing concentration of complexes **Pd1**–**Pd2** against BSA protein, suggestive of the formation of complex-BSA system. The emission intensities decrease considerably with the increasing concentration of the complexes (with no notable alteration in the emission peak position), and this could be attributed to the changes in the secondary structure of BSA causing the variation of microenvironment around BSA tryptophan (Manojkumar et al. 2019).

The change in the emission intensity fitted well into the Stern–Volmer equation (*K*_sv_ and *k*_q_) and Scatchard equations (*K*_*F*_ and n). The values of *k*_q_, *K*_*SV*_, *K*_*F*_, and n are provided in Table 3. The values of* K*_*SV*_ (8.78–25.53 × 10^4^ M^−1^) illustrate that the interactions of complexes **Pd1**–**Pd4** with the protein *i.e*. BSA is not fully controlled by diffusion (Milutinović et al. 2017). Similarly, the calculated *k*_*q*_ values (3.82–11.10 × 10^13^, M^−1^ s^−1^), are higher than most known quenchers (10^10^ M^−1^ s^−1^) approving static quenching mechanism (Poloni et al. 2019). Notably, the *K*_*F*_ values (1.10–10.01 × 10^3^ M^−1^) for **Pd1**–**Pd4** are within the optimum values of 10^3^–10^6^ M^−1^ (Topală et al. 2014), showing that the complexes can easily be transported and released to the target. The computed values of n (**≈** 1), reveal that only a single binding site of BSA is accessible for the interaction with the Pd(II) complexes. The BSA fluorescence quenching constants and binding constants, show strong and favourable binding of the complexes to the protein, and the order of binding is on par with the kinetic reactivity.

### Molecular docking

**Fig. 7:**
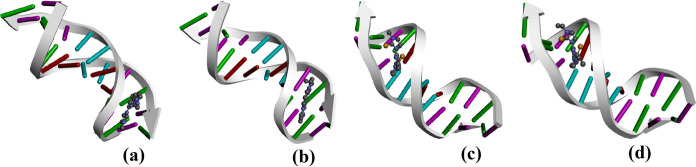
The best docked conformer of **Pd1** (**a**), **Pd2** (**b**), **Pd3** (**c**) and **Pd4** (**d**) with DNA duplex (scaled ball and stick diagrams), depicting intercalative binding

**Fig. 8:**
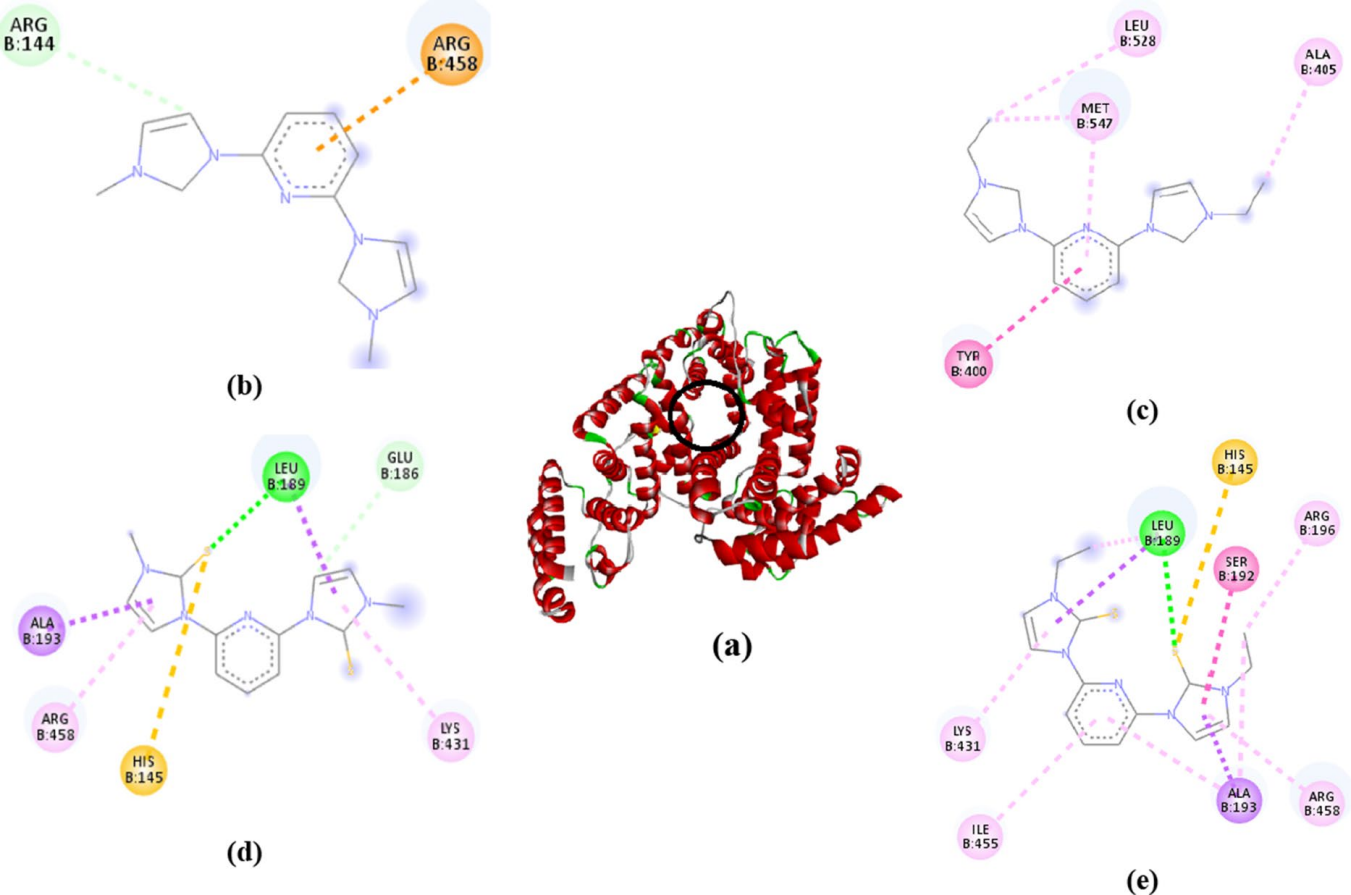
**a** BSA protein binding pocket; 2D interactions of **Pd1** (**b**), **Pd2** (**c**), **Pd3** (**d**), **Pd4** (**e**) on the active site of BSA protein

MD simulations were performed to elucidate the binding affinities, and interactions of Pd-complexes with DNA and BSA (Figs. 7 and 8). The complexes demonstrated favourable docked score of − 6.7 kcal/mol (**Pd1**) and –6.6 kcal/mol (**Pd2)** and –6.4 kcal/mol for both **Pd3 a**nd **Pd4** (Table S11), roughly matching the experimental observations (Table 2). Docked poses and nucleotides around Pd-complexes are depicted in Figs. 7 and S44. Nucleotides DC11 & 15 and DG 16 facilitate C–H bond interactions with **Pd1**. The stability of **Pd2** is contributed significantly by π-anion and C–H bond interactions with DG12 and DA18, respectively. Additionally, the pyrazolyl moiety in both **Pd3** and **Pd4** interacts with DT 7 nucleotide through π-anion interactions. A summary of the chemical interactions of **Pd1**-**Pd4** with DNA nucleotides is provided in **Table S12**. Consistent with the experimental trend, the docked models (Fig. 7) show intercalative binding mode.

The docked BSA protein energies are ordered as **Pd1** (– 6.9 kcal/mol) > **Pd2** (– 6.5 kcal/mol) and **Pd3** (– 6.7 kcal/mol**) ≈ Pd4** (– 6.8 kcal/mol), **Table S11**, nearly agreeing with the experimental trend (Table3). C–H bond and π-cation intermolecular interactions are formed between the pyridyl and pyrazolyl moieties of **Pd1** with ARG144 and ARG458 amino acids, respectively. TYR400, ALA405, and LEU528 residues form π–π T-shaped and π-alkyl (MET547) interactions with **Pd2**. Also, conventional H bond (LEU189), C–H bond (GLU186), π-sulfur (HIS145), π-alkyl (LYS431, ARG458 and ILE455) and π–σ (ALA193) interactions are pre-dominant in both **Pd3** and **Pd4**. A list of intermolecular interactions together with the active amino acids of **Pd1**-**Pd4** are given in **Table S12**.

## Conclusions

In summary, the syntheses and structural elucidation of Pd(II) complexes bearing CNC and SNS pincer-type ligands was achieved. The molecular structure of **Pd2** reveal a distorted square planar geometry. Kinetic reactivity of the Pd-complexes with the biological nucleophiles is controlled by both electronic and ring strain of the chelating ligands. DFT simulations of the complexes agree with the observed kinetic trends. The values of ΔH^≠^, ΔS^≠^ and ΔG^≠^_25 °C_ reveal an associative mode of substitution reactions. Isokinetic linear relationships support a single reaction pathway. Overall spectroscopic methods indicate intercalation binding mode, and the binding order matches kinetic lability. The energy calculations of the docked models show reasonable binding affinities of the Pd-complexes to both DNA and BSA. The models also show that the complexes are tightly anchored on the active sites of DNA and protein and confirmed the intercalating binding mode observed in the experimental studies.

## Supplementary data

The supplementary material contains the detailed procedures for ligand synthesis, X-ray crystallography analyses, DFT, electrochemical, stability, substitution kinetics, DNA/BSA, molecular studies, NMR, FT-IR spectroscopic spectral and mass spectral data. The material also contains DFT structures, CV voltammograms, substitution kinetics plots, UV-vis and fluorescence spectra, molecular diagrams and Tables. The crystallographic data entry for compound Pd2 is given by the deposition number CCDC 2179373.

### Supplementary Information

Below is the link to the electronic supplementary material.

Supplementary file 1 (DOCX 5480 KB)
