# Supplementary material for: Electronic and ring size effects of N-heterocyclic carbenes on the kinetics of ligand substitution reactions and DNA/protein interactions of their palladium(II) complexes
Source: Biometals. 2023 May 15;36(5):1109–23. doi: 10.1007/s10534-023-00507-8 (PMC10545578; doi:10.1007/s10534-023-00507-8)
Supplement: Supplementary file 1 — (DOCX 5480 KB) [file 10534_2023_507_MOESM1_ESM.docx]

**Electronic and ring size effects of N-heterocyclic carbenes on the kinetics of ligand substitution reactions and DNA/protein interactions of their palladium(II) complexes**

**Reinner O. Omondi. Deogratius Jaganyi. Stephen O. Ojwach**.

R O. Omondi

School of Chemistry and Physics, University of KwaZulu-Natal, Private Bag X01, Scottsville, Pietermaritzburg 3209, South Africa.

D. Jaganyi

School of Pure and Applied Sciences, Mount Kenya University, P.O. Box 342-01000, Thika, Kenya.

Department of Chemistry, Faculty of Applied Sciences, Durban University of Technology, P.O. Box 1334, Durban 4000, South Africa

SO. Ojwach*****

School of Chemistry and Physics, University of KwaZulu-Natal, Private Bag X01, Scottsville, Pietermaritzburg 3209, South Africa.

e-mail: Ojwach@ukzn.ac.za

# **1. Materials and instrumentation**

All manipulations of air and/or water-sensitive compounds were performed under a dry nitrogen atmosphere using standard Schlenk techniques. All solvents were analytical grade and used as obtained. Chemicals, 3-methylpyrazole (97%), diethylene glycol dimethyl ether (99.5%), potassium metal (98%), 2,6-dibromopyridine (98%), 1,3-dibromobenzene (97%), 1-methylimidazole (99%), silver(I) oxide (99.9%), palladium(II) dichloride (99%), silver tetrafluoroborate (98%), thiourea (99%), L-methionine (98%), guanosine 5′-diphosphate disodium salt (90%), tetrabutylammonium tetrafluoroborate (TBABF_4_) for electrochemical analysis (99%), tris(hydroxymethyl)aminomethane (99%), 32 wt. % hydrochloric acid (98%), thiourea (99%), L-methionine (98%), guanosine 5′-diphosphate disodium salt (90%), ethidium bromide (EB) (95.0%), calf thymus DNA (CT-DNA), and bovine serum albumin (BSA) (98%) were purchased from Merck and used without further purification. Ultrapure water (Modulab Systems) was used for all aqueous reactions and procedures.

NMR spectra were recorded on a Bruker Avance 300 and 500 MHz spectrometer in DMSO-D6 with residual signal of solvents as the internal standard at ambient temperature. Chemical shifts were reported in δ units. Coupling constants (*J*) were calculated in Hertz (HZ). Mass spectral analyses were acquired on a Shimadzu LC-MS 2020 Spectrometer or Waters TOF Micro-mass LCT Premier Spectrometer. Elemental analyses were performed on a Thermal Scientific Flash 2000 CHN analyser. Crystal data analyses were performed on a Bruker Apex Duo diffractometer equipped with an Oxford Instrument. Cary 100 Bio UV-visible spectrophotometer was used to determine a suitable wavelength for monitoring the substitution reactions. Applied Photophysics SX 20 stopped-flow reaction analyser coupled to an online acquisition system was used for kinetic studies reactions (thermo-controlled within ± 0.1 °C). Jenway 4330 pH/conductivity meter with a 4.5 mm micro-electrode was used to determine the pH measurements. Before use, the pH meter was calibrated with three standard buffer solutions: pH 4.0, 7.0 and 10.0.

# **2. Preparation of ligands**

## ***2.1 2,6-bis(3-methylimidazolium-1-yl)pyridine dibromide (1)***

A mixture of 2,6-dibromopyridine (3.20 g, 13.51 mmol) and 1-methylimidazole (2.15 mL, 27.11 mmol) was heated at 150 °C in the absence of solvent for 20 h to give an off-white solid. The solid was cooled to room temperature and washed with dichloromethane. Yield: 3.8 g (70%). ^1^H NMR (400 MHz, DMSO-d6): δ_H_ (ppm): 4.06 (s, 6H, CH_3_); 8.07 (s, 2H, imidazole, CH); 8.26 (d, ^3^J_HH_ = 8.1, 2H, pyridine, CH); 8.60 (t, ^3^J_HH_ = 8.1, H, pyridine, CH); 8.81 (s, 2H, imidazole, CH); 10.63 (s, 2H, imidazole, CH). ^13^C NMR (DMSO-d_6_): δ_C_ (ppm): 36.50 (CH_3_); 114.06 (imidazole, CH); 119.07 (pyridine, CH); 124.95 (imidazole, CH); 136.28 (imidazole, CH); 144.20 (pyridine, CH); 145.85 (pyridine, C). FT-IR (cm^-1^): υ(C-H, aromatic) = 3365; υ(C-H, alkyl) = 3017; υ(C= N, imidazole) = 1605; υ(C=N, pyridine) = 1531; υ(C-N, imidazole) = 1077. LC MS/ESI^+^, *m/z (*%) = 240 [M^+^, -3H, 100]; 241 [M^+^,-2H, 10].

## ***2.2 2,6-bis(3-ethylimidazolium-1-yl)pyridine dibromide (2)***

The ligand was prepared following the synthetic protocol described for **1**, using 2,6-dibromopyridine (3.20 g, 13.51 mmol), and 1-ethylimidazole (2.61 mL, 27.11 mmol). Brown solid. Yield: 4.32 g (74%). ^1^H NMR (400 MHz, DMSO-d6): δ_H_ (ppm): 1.57 (t, ^3^J_HH_ = 7.2, 6H, CH_3_); 4.42 (dd, ^3^J_HH_ = 7.2, 4H, CH_2_); 8.22 (s, 2H, imidazole, CH); 8.31 (d, ^3^J_HH_ = 8.0, 2H, pyridine); 8.61 (t, ^3^J_HH_ = 8.0, 1H, pyridine, CH); 8.89 (s, 2H, imidazole, CH); 10.77 (s, 2H, imidazole, CH).  ^13^C NMR (DMSO-d_6_): δ_C_ (ppm): 14.49 (CH_3_); 45.08 (CH_2_); 114.12 (imidazole, CH); 119.35 (pyridine, CH); 123.47 (imidazole, CH); 135.60 (imidazole, CH); 144.75 (pyridine, CH); 145.24 (pyridine, C). FT-IR (cm^-1^): υ(C-H, aromatic) = 3342 υ(C-H, alkyl ) = 3067; υ(C= N, imidazole) = 1609; υ(C=N, pyridine) = 1531; υ(C-N, imidazole) = 1078.

## ***2.3 2,6-bis(3-methylimidazole-2-thione)pyridine (3)***

A mixture of **1** (2.0 g, 5.00 mmol), K_2_CO_3_ (1.41 g, 10.20 mmol), S_8_ powder (0.33 g, 10.20 mmol) was refluxed in dry methanol (40 mL) for 8 h after which the solvent was evaporated, and the residual solid was extracted with dichloromethane. The dichloromethane extract was concentrated to 4 mL and 1 mL of methanol was added to yield a white crystalline solid. Yield: 1.11 g (73%). ^1^H NMR (400 MHz, DMSO-d_6_): δ_H_ (ppm): 3.57 (s, 6H, CH_3_); 7.39 (d, ^3^J_HH_ = 2.6, 2H, imidazole, CH); 7.81 (d, ^3^J_HH_ = 2.6, 2H, imidazole, CH); 8.22 (t, ^3^J_HH_ = 8.0, 1H, pyridine, CH); 8.72 (d, ^3^J_HH_ = 8.0, 2H, pyridine, CH). ^13^C NMR (DMSO-d_6_): δ_C_ (ppm): 34.58 (CH_3_); 116.11 (pyridine, CH); 116.64 (imidazole, CH); 119.83 (imidazole, CH); 140.06 (pyridine, CH); 148.30 (pyridine, C); 161.79 (C =S). FT-IR (cm^-1^): υ(C-H, aromatic) = 3160; υ(C-H, alkyl) = 3078; υ(C= N, imidazole) = 1689; υ(C=N, pyridine) = 1575; υ(C=S, imidazole) = 1142, υ(C-N, imidazole) = 1040. LC MS/ESI^+^, *m/z (*%) = 303 [M^+^, 25]; 326 [M^+^,+ Na, 100]; 377 [M^+^,+ 3Na, 28]; 629 [2M^+^,+ Na, 5].

## ***2.4 2,6-bis(3-ethylimidazole-2-thione)pyridine (4)***

The ligand was synthesised in a similar fashion to **2**, using **3** (2.16 g, and 5.01 mmol), K_2_CO_3_ (1.41 g, 10.21 mmol), S_8_ (0.33 g, 10.21 mmol). White crystalline solid. Yield: 1.26 g (75%). ^1^H NMR (400 MHz, DMSO-d_6_): δ_H_ (ppm): 1.30 (t, ^3^J_HH_ = 7.2, 6H, CH_3_); 4.09 (dd, ^3^J_HH_ = 7.2, 4H, CH_2_); 7.43 (d, ^3^J_HH_ = 2.6, 2H, imidazole, CH); 7.81 (d, ^3^J_HH_ = 2.6, 2H, imidazole); 8.20 (t, ^3^J_HH_ = 8.0, 1H, pyridine, CH); 8.69 (d, ^3^J_HH_ = 8.0, 2H, pyridine, CH). ^13^C NMR (DMSO-d_6_): δC (ppm): 13.62 (CH_3_); 41.95 (CH_2_); 116.61 (pyridine, CH); 117.01 (imidazole, CH); 118.31 (imidazole, CH); 139.92 (pyridine, CH); 148.29 (pyridine, C); 160.98 (C =S). FT-IR (cm^-1^): υ(C-H, aromatic) = 3397; υ(C-H, alkyl) = 3097; υ(C= N, imidazole) = 1645; υ(C=N, pyridine) = 1449; υ(C=S, imidazole) = 1117, υ(C-N, imidazole) = 992. LC MS/ESI^+^, *m/z (*%) = 332 [M^+^, + H, 100]; 354 [M^+^, + Na, 100]; 685 [2M^+^,+Na, 18].

# **3. X-ray crystallography**

X-ray data were recorded on a Bruker Apex Duo diffractometer equipped with an Oxford Instruments Cryojet operating at 100(2) K and an Incoatec microsource operating at 30 W power. The data were collected with Mo Kα (λ = 0.71073 Å) radiation at a crystal-to-detector distance of 50 mm. The following conditions were used for the data collection: omega and phi scans with exposures taken at 30 W X-ray power and 0.50º frame widths using APEX2 (APeX 2010). The data were reduced with the programme SAINT (Sheldrick 2015) using outlier rejection, scan speed scaling, as well as standard Lorentz and polarisation correction factors. A SADABS semi-empirical multi-scan absorption correction was applied to the data. Direct methods, SHELXS-2014 andWinGX (Farrugia 2012), were used to solve all three structures. All non-hydrogen atoms were in the difference density map and refined anisotropically with SHELXL-2014. All hydrogen atoms were included as idealised contributors in the least squares process. Their positions were calculated using a standard riding model with C-H_aromatic_ distances of 0.93 Å and Uiso= 1.2 Ueq, C–H_methylene_ distances of 0.99 Å and Uiso = 1.2 Ueq and C–H_methyl_ distances of 0.98 Å and Uiso= 1.5 Ueq.

# **4. Density functional theoretical calculations**

All geometry optimisation and energy computations were performed without imposing any symmetry restriction using Density Functional Theory (DFT) with the B3LYP level of theory (Frisch et al. 2010), adopting the LANL2DZ basis set. Solvent effect was examined *via* single point calculations using conductor-like polarisable continuum implicit solvent formalism (Cossi et al. 2003). The complexes were modelled in water media. To study the electronic properties of the complexes, quantum chemical descriptors such as the values of the highest occupied molecular orbital energy (E_HOMO_) and the lowest unoccupied molecular orbital energy (E_LUMO_), chemical hardness (ɳ), chemical softness (σ), global electrophilicity indices (ω), nucleophilicity (ε) and dipole moments were calculated. Natural bond orbitals (NBO) analysis was adopted to compute the atomic charges of the selected atoms in the complexes at the same theoretical level. Gaussian 09W programme suite was employed to visualise geometry optimised structures, and to calculate the minimum energy of the structure. GaussView 5.0 was utilised to prepare the input files.

# **5. Electrochemical studies of the palladium complexes**

Electrochemical studies were performed in oxygen-free solutions of Pd-complexes in DMSO consisting of 0.1 M equivalents of TBABF_4_ as a supporting electrolyte. All electrochemical experiments were conducted at 25.0 ± 0.2 °C. The voltammograms were obtained in a potential window of -2.0 to +2.0 V. Cyclic voltammetry (CV) studies of the complexes were performed at different potential scan rates ranging from 25 - 250 mVs^-1^. Contrarily, square wave voltammetry (SWV) was carried out at a set potential of 100 mVs^-1^.

# **6. Stability of the complexes in aqueous and DMSO solutions**

The stabilities of the complexes (1.0 x 10^4^ M) in aqueous solutions, Tris-HCl buffer (pH = 7.2) or DMSO were qualitatively explored by ^I^H NMR and UV-Vis spectroscopies. The electronic spectral changes were recorded over a period of 24 h (in the case of water exchange kinetics) or 72 h (for complex stability in DMSO media), at room temperature.

# **7. Substitution kinetics with biomolecules**

Substitution reactions were monitored at physiological conditions (pH 7.2) in the presence of 50 μM Tris-HCl buffer (pH = 7.2), with the addition of 50 mM NaCl to prevent any spontaneous hydrolysis of the complexes. Freshly prepared stock solutions of the nucleophiles approximately 50-fold excess of the concentration of the complex were serially diluted with the aqua solution to afford 40, 30, 20, and 10-fold excesses of the complex concentration to maintain pseudo-first order conditions. The reactions were performed using stopped-flow spectrophotometry at appropriate working wavelengths (pre-established from the UV-Vis spectra) and were followed for at least eight half-lives. Concentration dependence studies were performed at a constant temperature of 298 K. Kinetic traces were fitted into a non-linear least square fit to generate pseudo-first-order rate constants (*k*_obs_) using equation (S1) (Tobe, Burgess 1999).

A_t_ = Ao + (Ao-A∞)exp(-*kobs*t) (S1)
where At = absorbance at time t, A_o_ = absorbance at the onset of the reaction, A∞ = absorbance
at the end of the reaction. The second-order rate constants (*k*_2_) were derived from the dependence of the observed pseudo-first-order rate constant, *k*_obs_, on the concentration of the incoming nucleophile, [Nu], using equation (S2) (Atwood 1997).

*k*_obs_ = *k*_2_[Nu] + 𝑘_−2_ (S2)

where 𝑘_−2_ is the first-order rate constant for the reverse reaction. The values of *k_2_* and *k_-2_* were obtained from the slope and y-intercept of the plot, respectively. A plot with a zero y-intercept means that the forward reaction is irreversible, then the relationship between *k_obs_* and [Nu] can be illustrated by equation (S3), while one with an appreciable y-intercept implies that reaction proceeds in a reversible manner or through the coordination of a solvent molecule (solvotic pathway).

*k*_obs_ =*k*_2_[Nu] (S3)

Temperature dependence reactions were systematically observed over a range of 298-318 K at an interval of 5 K. The activation parameters were determined from Eyring equation (S4) (Atwood 1997).

In (*k*_2_/T) = - ΔH^≠^/RT + (23.8 + ∆*S*^≠^/R) (S4)

where ΔH^≠^, ∆*S*^≠^, T and are activation enthalpy, activation entropy, temperature, and gas constant, respectively.

The Gibbs energy of activation (ΔG^≠^), which consists of ∆*S*^≠^ and ΔH^≠^, was calculated from equation (S5) (Eyring 1935, Gray, Olcott 1962).

ΔG^≠^ = ΔH^≠^ - T∆*S*^≠^ (S5)

where T is temperature in kelvin

# **8. CT-DNA and BSA binding studies**

## ***8.1 UV–Visible absorption spectroscopy***

UV-visible absorption titration of CT-DNA binding was performed following our previously reported procedures (Omondi et al. 2020a, Omondi et al. 2021). The concentration of CT-DNA in Tris-HCl buffer was spectrophotometrically determined at coefficient *ɛ*_260_ = 6600 mol^−1^ cm^−1^. The purity of CT-DNA solution was determined from the ratio of electronic absorption at the wavelength of 260 nm. The stock solution of the Pd-complexes was made using ultrapure water. A constant concentration of the Pd-complexes was titrated with the increasing amounts of CT-DNA stock solution. The Pd-CT-DNA solutions were incubated for 8 min at room temperature before collecting the UV-Vis spectra from the region 230-500 nm. The intrinsic binding constant, *K*_b_, was obtained from Wolfe-Shimer equation (S6).

[DNA]/(ε_a_-ε_f_) = [DNA]/(ε_b_-ε_f_)+1/(*K*_b_(ε_b_-ε_f_)) (S6) where [DNA] is the concentration of CT-DNA in base pairs; ε_a_ is the apparent absorption coefficient at a given [DNA]; ε_b_ is the extinction coefficient of the fully bound metal complex to DNA, and ε_f_ is the extinction coefficient of the free metal complex in solution. The *K*_b_ values were determined from the ratio of the slope to the y-intercept in plots of [DNA]/(ε_a_ - ε_f_) *vs* [DNA]. The standard Gibb’s free energy (∆G) values were obtained following van't Hoff equation (S7).

∆G = – RT ln *K_b._* (S7)

## ***8.2 DNA fluorescence spectroscopic studies***

The fluorescence quenching experiment with EB-DNA system was also carried out based on our previously published procedures (Omondi et al. 2020b). EB-DNA composite was prepared in Tris-HCl buffer solution and kept in the dark overnight for the experiment. The fluorescence emission was exited at 525 nm, and the emission recorded from 530 nm to 700 nm at room temperature.

The Stern-Volmer binding constant (*K_SV_*) and the bimolecular quenching rate constant (*K_q_*) were calculated from the classical Stern-Volmer equation (S8).

*I*_o_/*I* = 1 + *K*_sv_[Q] = 1 + *k*_q_τ_0_[Q] (S8) where *I*_o_ and *I* are the emission intensities in the absence and presence of the quencher, respectively; [Q] is the concentration of the quencher and τ_0_ is the average lifetime of the fluorophore (10^-8^ s) without the quencher.

The apparent association constant, *K*_app_, was obtained from the equation (S9).

*K*_EB_[EB] = *K*_app_[Q] (S9) where [Q] is the quencher concentration leading to 50% reduction in the intensity of fluorescence EB-CT-DNA, *K*_EB_ = 10^7^ M^-1^. The values of the binding constant, *K*_F_, and the number of binding sites (n) were computed from Scatchard equation (S10).

log(*I*_o_ - *I*) / *I* = log*K*_F_ + *n* log[Q] (S10)

## ***8.3 BSA fluorescence spectral studies***

BSA fluorescence examinations were conducted according to our literature procedures (Omondi et al. 2021). The concentration of the BSA in Tris-HCl buffer was spectrophotometrically determined at coefficient *ɛ*_280_ = 44300 mol^−1^ cm^−1^. The concentration of BSA protein was constant (3 μM), and the quencher was added incrementally (0-120 μM) at room temperature. The spectra were recorded from 300-450 nm (with an excitation wavelength of 280 nm) after 10 min incubation at room temperature.

## ***8.4 Filter effect corrections***

Data correction was employed to the spectrophotometric titrations to compensate for the existing primary and/or secondary inner filter effects following literature procedures (Parker, Rees 1962) using equation (S11).

F_corr_ = F_obs_10(A_ex_+A_em_)/2 (S11)

where F_corr_ and F_obs_ are designated as the corrected and observed fluorescence intensities, respectively, whereas A_ex_ and A_em_ are the absorbance values at the excitation and emission wavelengths, respectively.

**9*. In silico* approach**

Molecular docking was carried using AutoDock Vina software suite (Trott, Olson 2010). The crystal structures of DNA (PDB ID: 1Z3F) and BSA (PDB ID:4F5S) were retrieved from protein data bank (<http://www.rcsb.org/>) at a solution of 1.60 and 2.47 A, respectively. The structures were refined by removing hetero atoms and water molecules. Polar hydrogen atoms as well as Kollman charges were added to the structures. The geometry optimisation of the Pd-complexes was performed by DFT method employing B3LYP functions. The ligand (i.e., complexes) and receptor were prepared using AutoDock Tools. BIOVIA Discovery Studio Visualizer 2022 package was used to produce molecular animations, images, and atomic interaction measurements.

# **10. ^1^H NMR spectra of ligands**

| 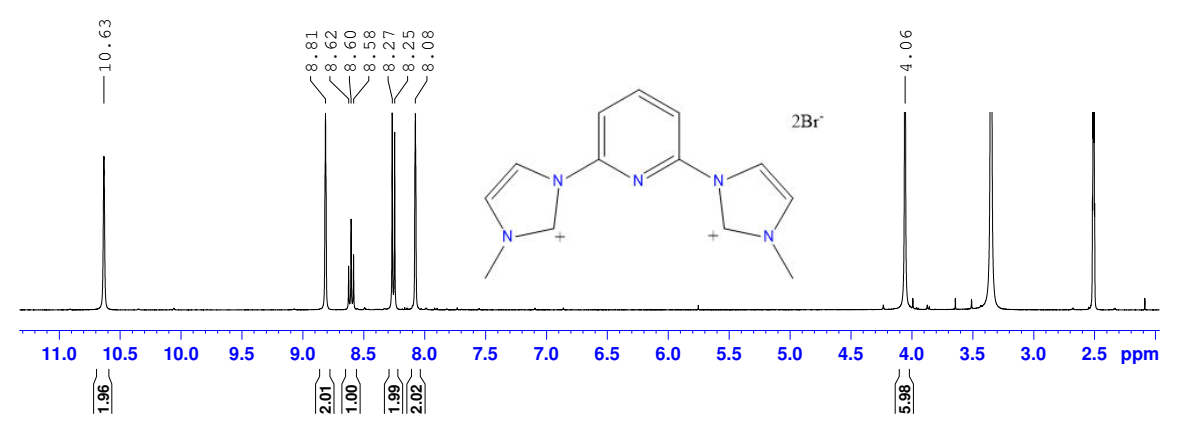 |
| --- |

**Fig. S1**: ^1^HNMR spectrum of **1**, δ_H_ (ppm): 4.06 (s, 6H, CH3); 8.07 (s, 2H, imidazole, CH); 8.26 (d, 2H, pyridine, CH); 8.60 (t, H, pyridine, CH); 8.81 (s, 2H, imidazole, CH); 10.63 (s, 2H, imidazole, CH).

| 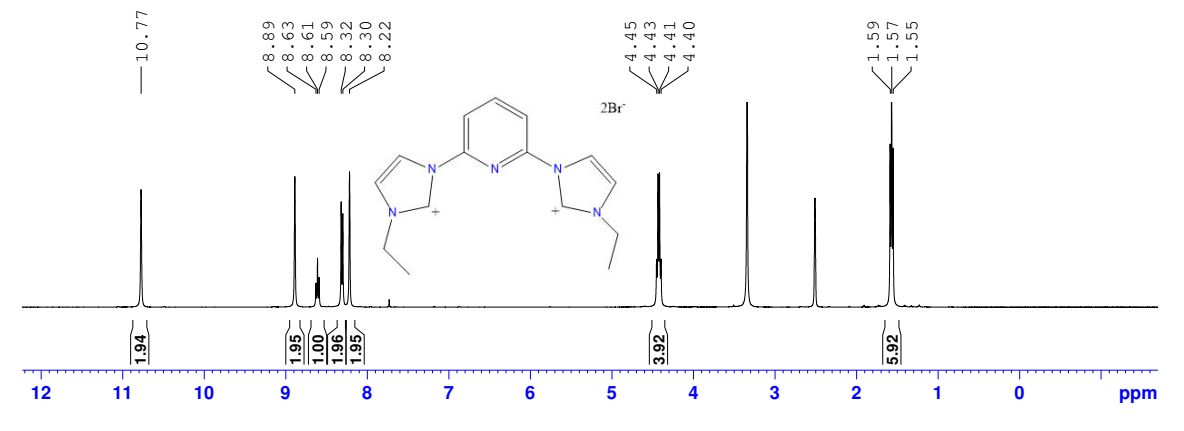 |
| --- |

## **Fig. S2**: ^1^HNMR spectrum of **2**, δ_H_ (ppm): 1.57 (t, 6H, CH_3_); 4.42 (dd, 4H, CH_2_); 8.22 (s, 2H, imidazole, CH); 8.31 (d, 2H, pyridine); 8.61 (1H, pyridine, CH); 8.89 (s, 2H, imidazole, CH); 10.77 (s, 2H, imidazole, CH).

| 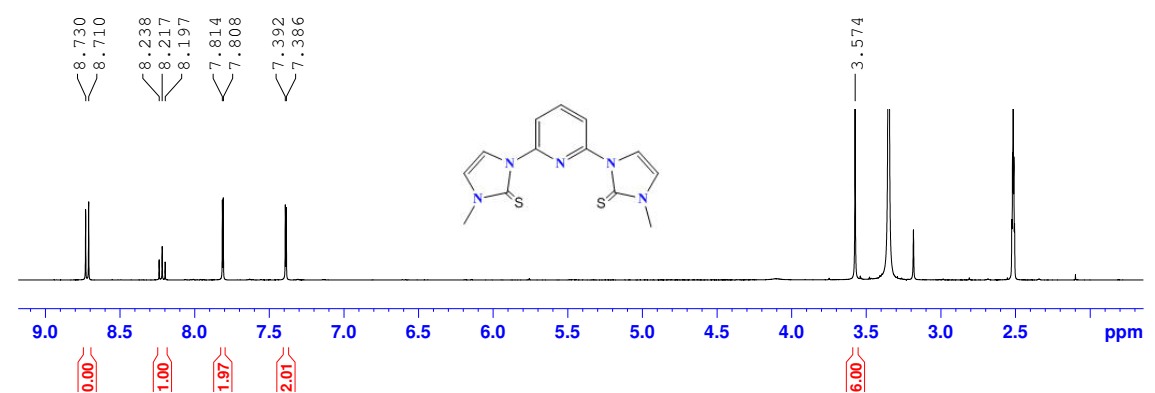 |
| --- |

## **Fig. S3**: ^1^HNMR spectrum of **3**, δ_H_ (ppm): 3.57 (s, 6H, CH_3_); 7.39 (d, 2H, imidazole, CH); 7.81 (d, 2H, imidazole, CH); 8.22 (t, 1H, pyridine, CH); 8.72 (d, 2H, pyridine, CH).

| 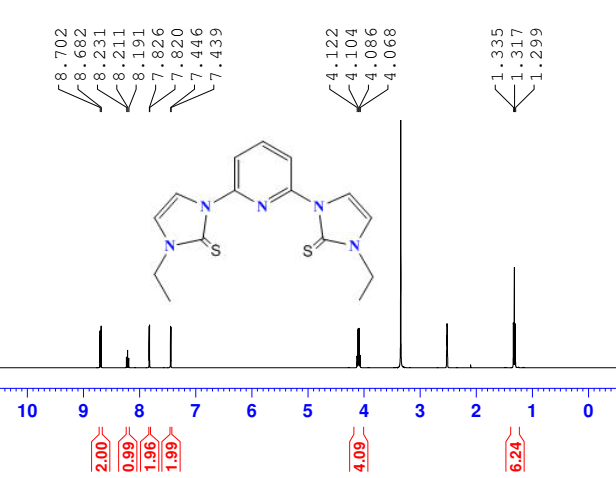 |
| --- |

**Fig. S4**: ^1^HNMR spectrum of **4**, δ_H_ (ppm): 1.30 (t, 6H, CH3); 4.09 (dd, 4H, CH2); 7.43 (d, 2H, imidazole, CH); 7.81 (d, 2H, imidazole); 8.20 (t, 1H, pyridine, CH); 8.69 (d, 2H, pyridine, CH).

| 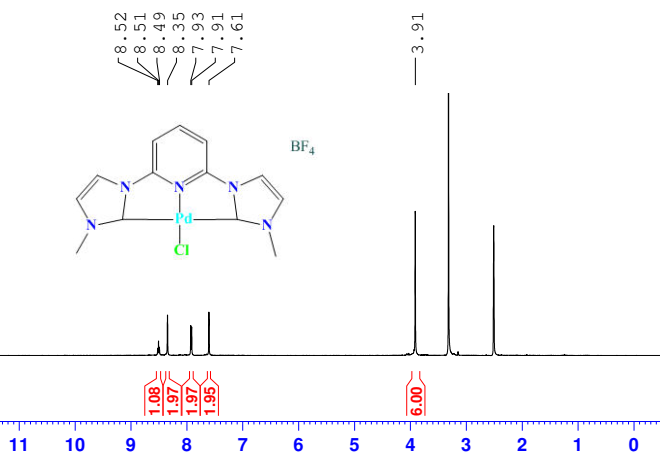 |
| --- |

**Fig. S5**: ^1^HNMR spectrum of **Pd1**, δ_H_ (ppm): 3.91 (s, 6H, CH3); 7.61 (s, 2H, imidazole); 7.92 (d, 2H, pyridine); 8.35 (s, 2H, imidazole); 8.51 (t, 1H, pyridine). The disappearance of the imidazolium CH proton at 10.63 ppm (**1**) upon complexation shows coordination of the CH moiety to the Pd(II) atom.

| 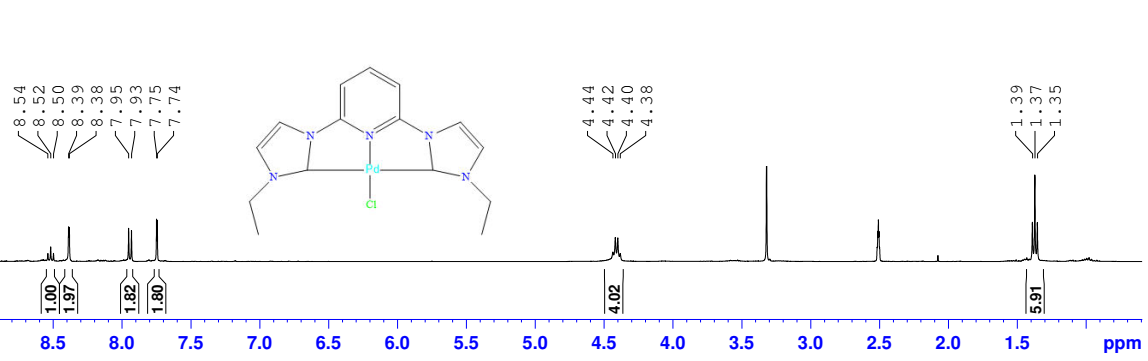 |
| --- |

## **Fig. S6**: ^1^HNMR spectrum of **Pd2,** δ_H_ (ppm): 1.37 (t, 6H, CH_3_); 4.41 (dd, 4H, CH_2_); 7.75 (d, 2H, imidazole); 7.94 (d, 2H, imidazole); 8.38 (d, 2H, pyridine); 8.52 (t, 1H, pyridine). The disappearance of the imidazolium CH proton (**2**) upon complexation indicate coordination of the CH moiety to the Pd(II) atom.

| 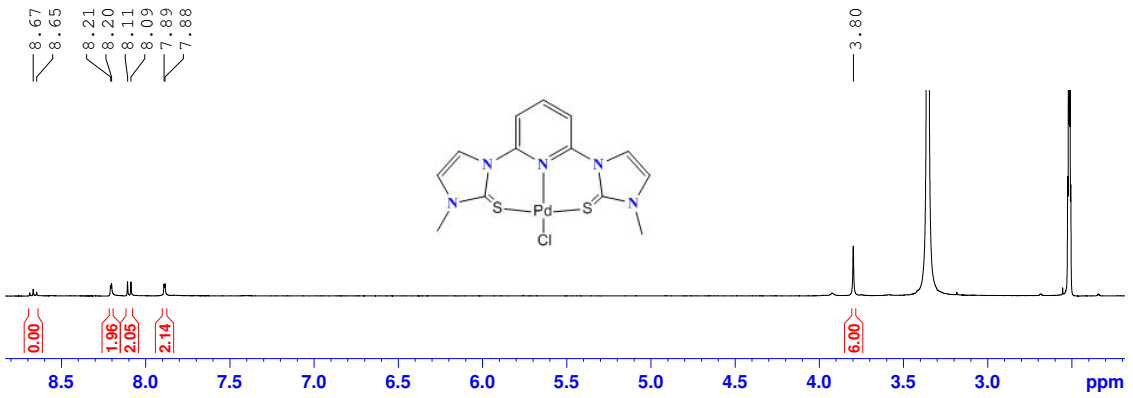 |
| --- |

## **Fig. S7:** ^1^HNMR spectrum of **Pd3**, with δ_H_ (ppm): 3.80 (6H, CH_3_); 7.85 (d, 2H, imidazole, CH); 8.10 (d, 2H, imidazole, CH); 8.20 (d, 1H, pyridine, CH); 8.66 (t, 1H, pyridine, CH).

| 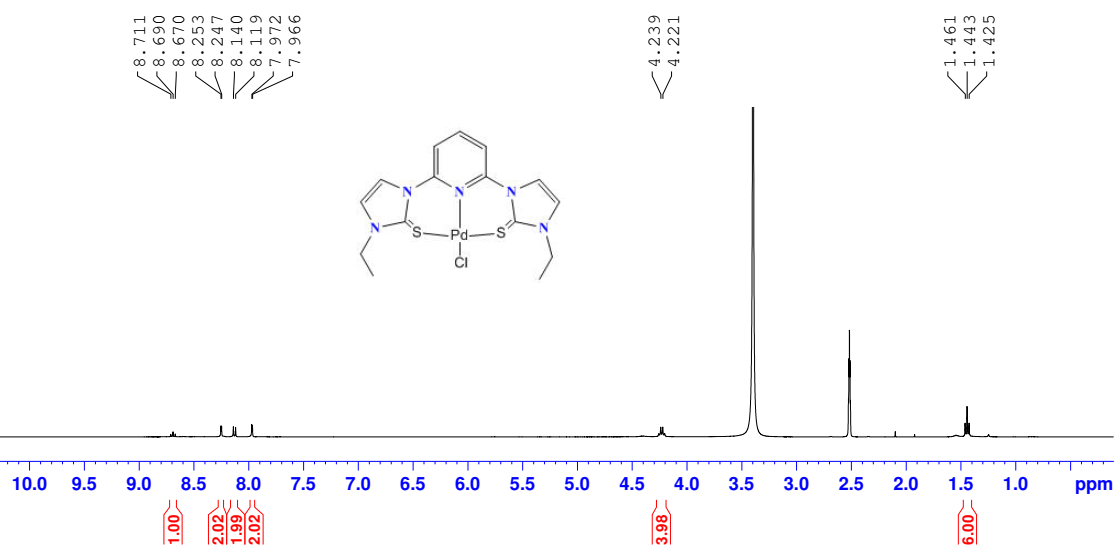 |
| --- |

## **Fig. S8**: ^1^HNMR spectrum of **Pd4,** δ_H_ (ppm): 1.44 (t, 6H, CH_3_); 4.23 (d, CH_2_); 7.97 (d, 2H, imidazole, CH); 8.13 (d, 2H, imidazole, CH); 8.25 (d, 2H, pyridine, CH); 8.70 (t, 1H, pyridine, CH).

| 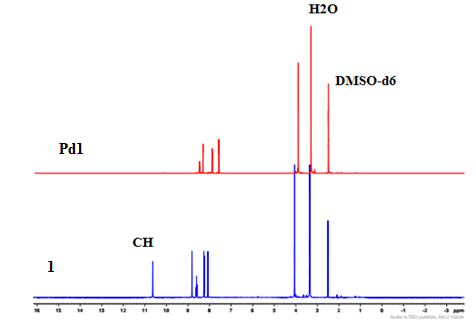 |
| --- |

**Fig. S9**: Overlays of ^1^H NMR spectra of **1** in DMSO-d6, and the respective complex **Pd1**, showing disappearance of the CH protons at 10.63 ppm (**1**) upon complexation (**Pd1**).

# **11. ^13^ C NMR spectra**

| 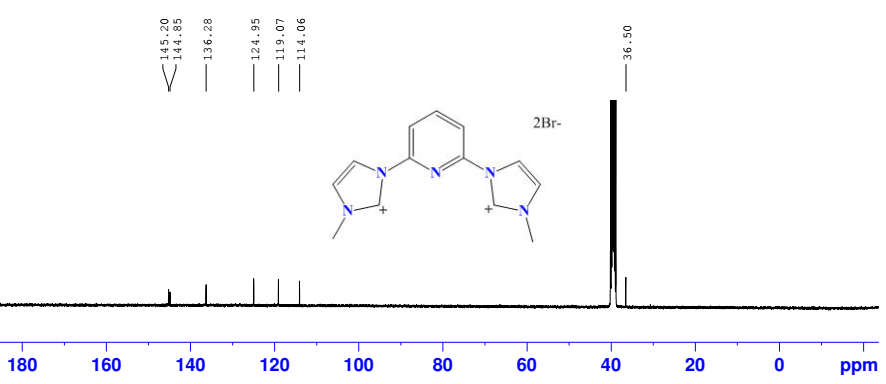 |
| --- |

## **Fig. S10:** ^13^C NMR spectrum of **1**, δ_C_ (ppm): 36.50 (CH_3_); 114.06 (imidazole, CH); 119.07 (pyridine, CH); 124.95 (imidazole, CH); 136.28 (imidazole, CH); 144.85 (pyridine, CH); 145.20 (pyridine, C).

| 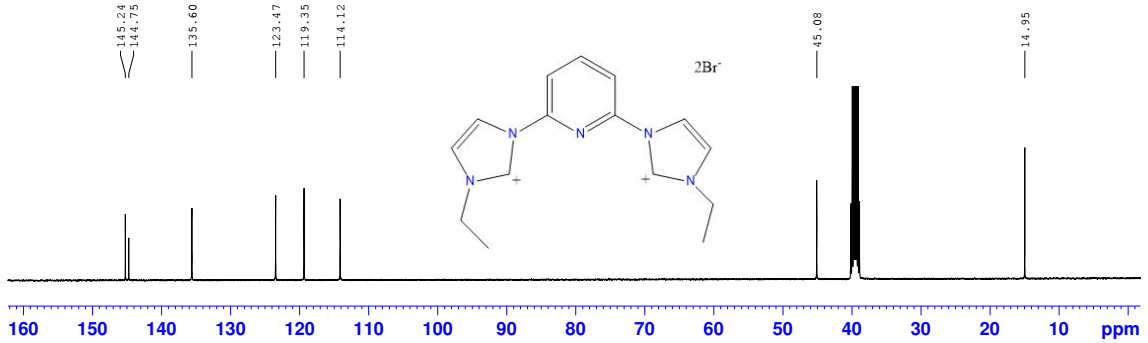 |
| --- |

## **Fig. S11:** ^13^C NMR spectrum of **2**, δ_C_ (ppm): 14.95 (CH_3_); 45.08 (CH_2_); 114.12 (imidazole, CH); 119.35 (pyridine, CH); 123.47 (imidazole, CH); 135.60 (imidazole, CH); 144.75 (pyridine, CH);

## 145.24 (pyridine, C)

| 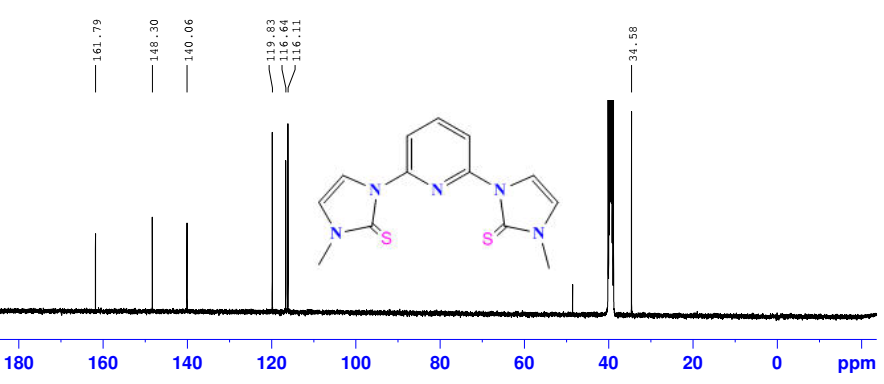 |
| --- |

## **Fig. S12:** ^13^C NMR spectrum of **3,** with δ_C_ (ppm): 34.58 (CH_3_); 116.11 (pyridine, CH); 116.64 (imidazole, CH); 119.83 (imidazole, CH); 140.06 (pyridine, CH); 148.30 (pyridine, C); 161.79 (C =S).

| 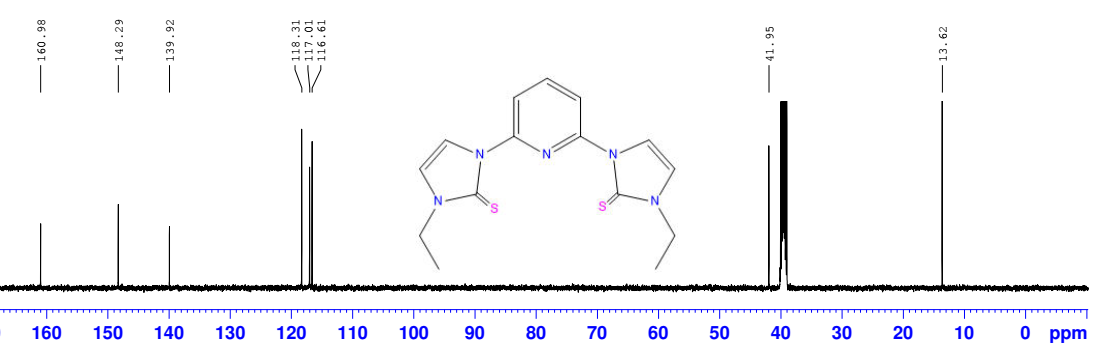 |
| --- |

## **Fig. S13:** ^13^C NMR spectrum of **4** with δ_C_ (ppm): 13.62 (CH_3_); 41.95 (CH_2_); 116.61 (pyridine, CH); 117.01 (imidazole, CH); 118.31 (imidazole, CH); 139.92 (pyridine, CH); 148.29 (pyridine, C); 160.98 (C =S).

| 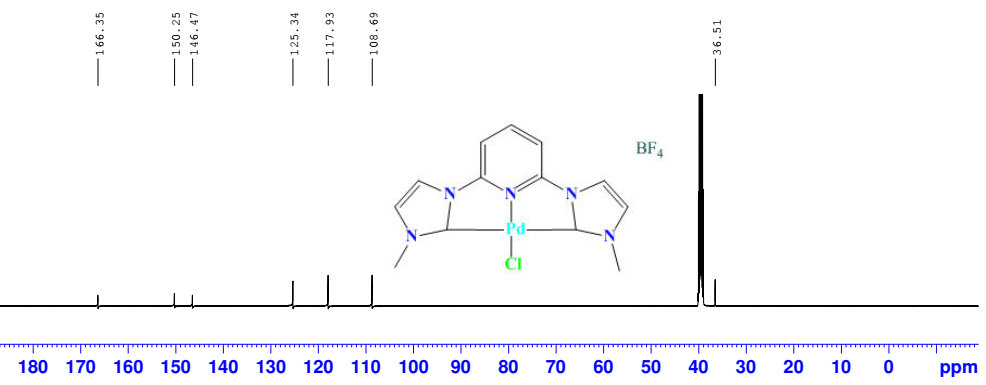 |
| --- |

## **Fig. S14:** ^13^C NMR spectrum of **Pd1**, δ_C_ (ppm): 36.51 (CH_3_); 108.69 (imidazole, CH); 117.93 (pyridine, CH); 125.34 (imidazole, CH); 146.47 (pyridine, CH); 150.25 (pyridine, CH); 166.35 (pyridine, C). The downfield shifts of the resonance of the carbene carbon at 145.20 ppm (**1**) to 166.35 pm (**Pd1**).

| 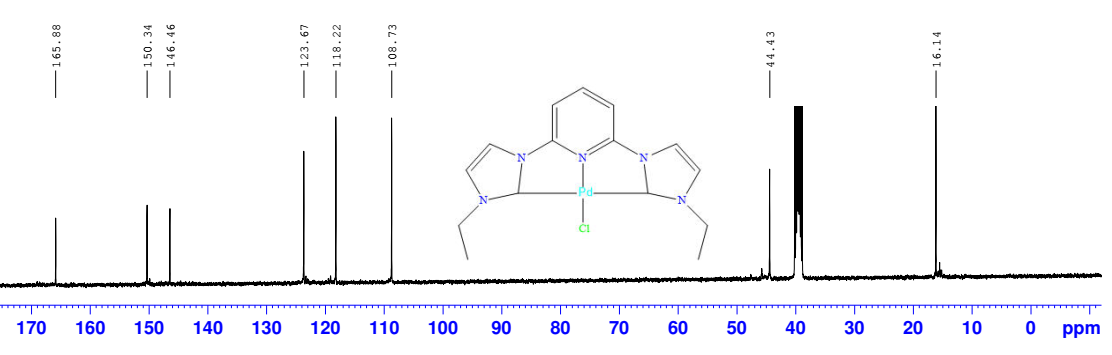 |
| --- |

## **Fig. S15****:** ^13^C NMR spectrum of **Pd2,** δ_C_ (ppm): 16.14 (CH_3_); 44.43 (CH_2_); 108.73 (imidazole, CH); 118.22 (pyridine, CH); 123.67 (imidazole, CH); 146.46 (pyridine, CH); 150.34 (pyridine, CH); 165.88 (pyridine, C). The downfield shifts of the resonance of the carbene carbon at 145.24 ppm (**1**) to 166.88 pm (**Pd2**) shows coordination.

| 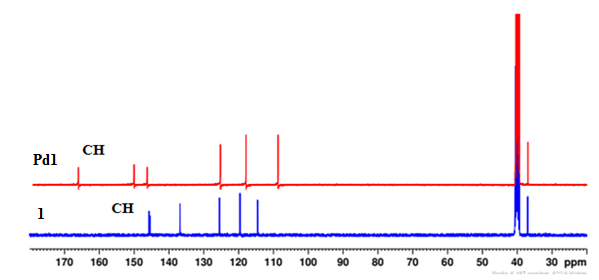 |
| --- |

## **Fig. 16**: Overlays of ^13^C NMR spectra of **1** in DMSO-d6, and the respective complex **Pd1**, displaying downfield shifts of the resonance of the carbene carbon at 145.20 ppm (**1**) to 166.35 pm (**Pd2**).

# **12. FT-IR spectra**

| 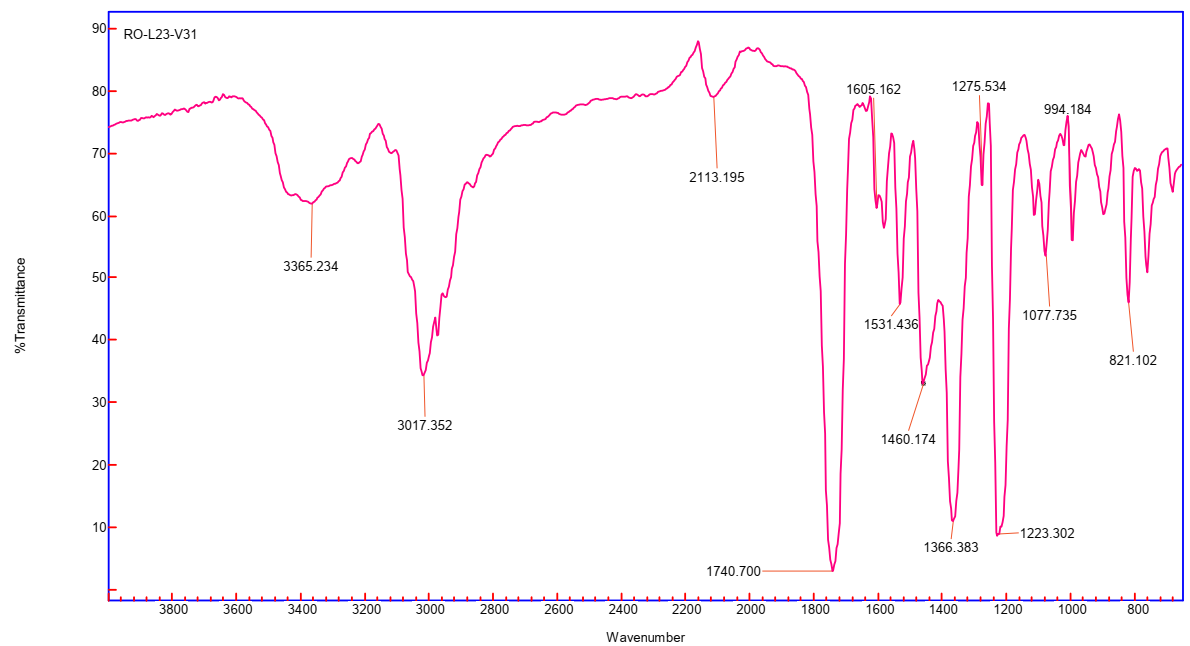 |
| --- |

**Fig. S17:** FT-IR spectrum of **1**, with υ(C-H, aromatic) = 3365; υ(C-H, alkyl) = 3017; υ(C= N, imidazole) = 1605; υ(C=N, pyridine) = 1531; υ(C-N, imidazole) = 1077. The strong and sharp absorption band at 1740cm^-1^ is attributed to the carbonyl stretching frequency for the acetone solvent impurity.

| 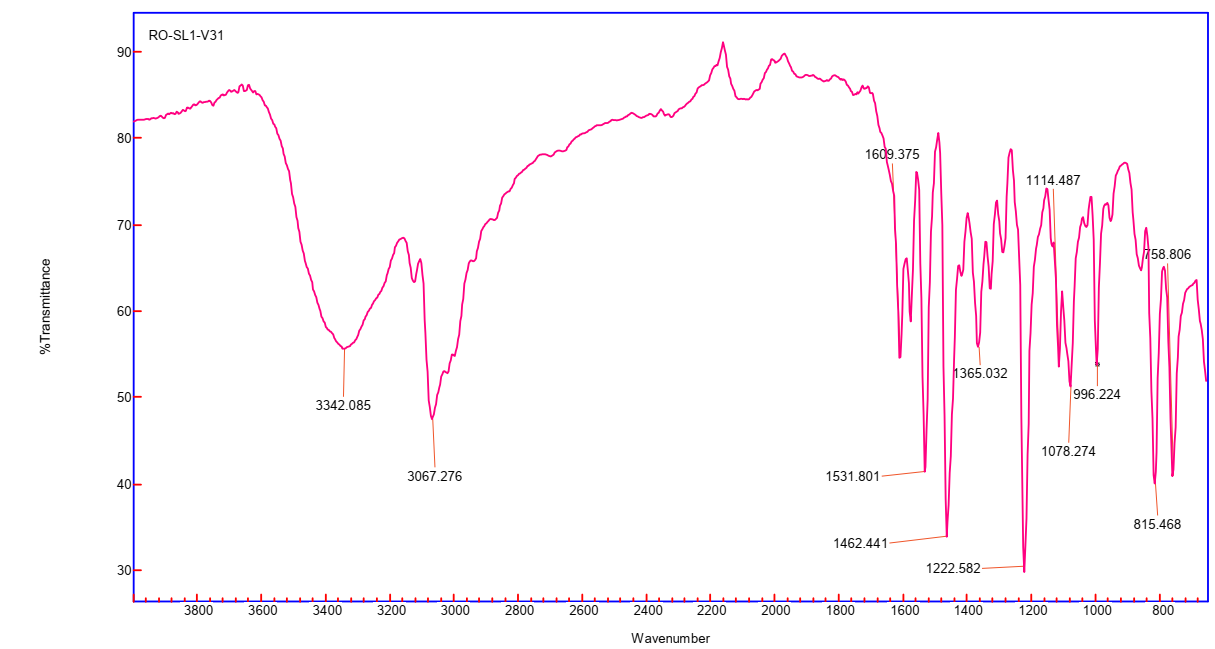 |
| --- |

**Fig. S18:** FT-IR spectrum of **2** with υ(C-H, aromatic) = 3342 υ(C-H, alkyl ) = 3067; υ(C= N, imidazole) = 1609; υ(C=N, pyridine) = 1531; υ(C-N, imidazole) = 1078.

| 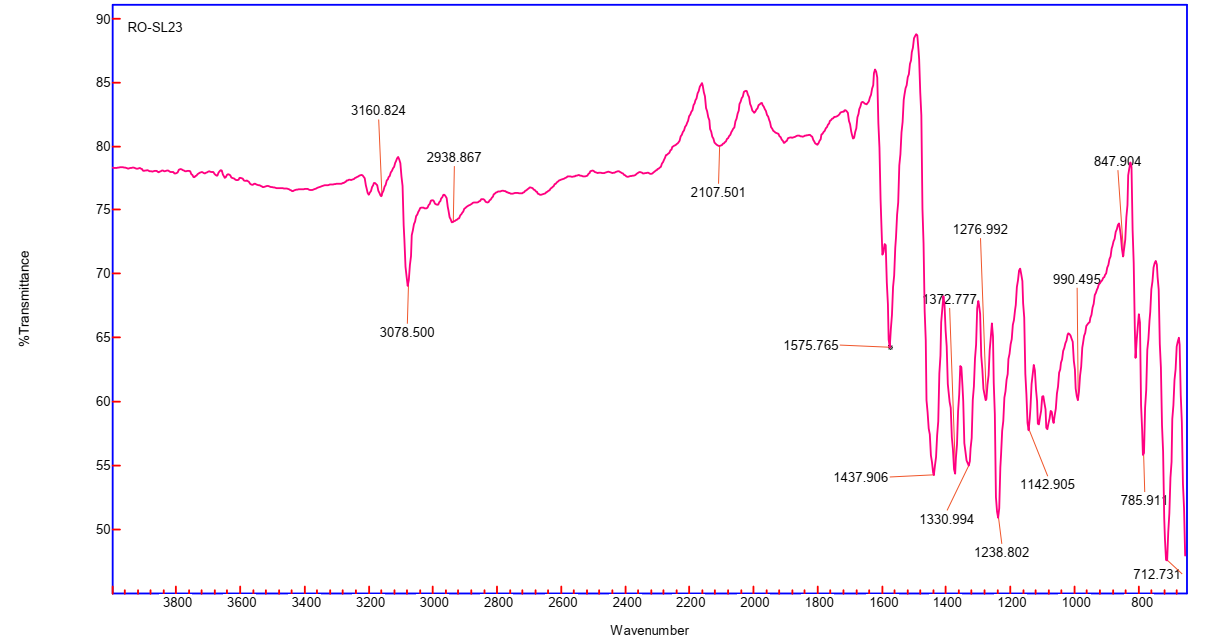 |
| --- |

## **Fig. S19:** FT-IR spectrum of **3** with υ(C-H, aromatic) = 3160; υ(C-H, alkyl) = 3078; υ(C= N, imidazole) = 1689; υ(C=N, pyridine) = 1575; υ(C=S, imidazole) = 1142, υ(C-N, imidazole) = 1040.

| 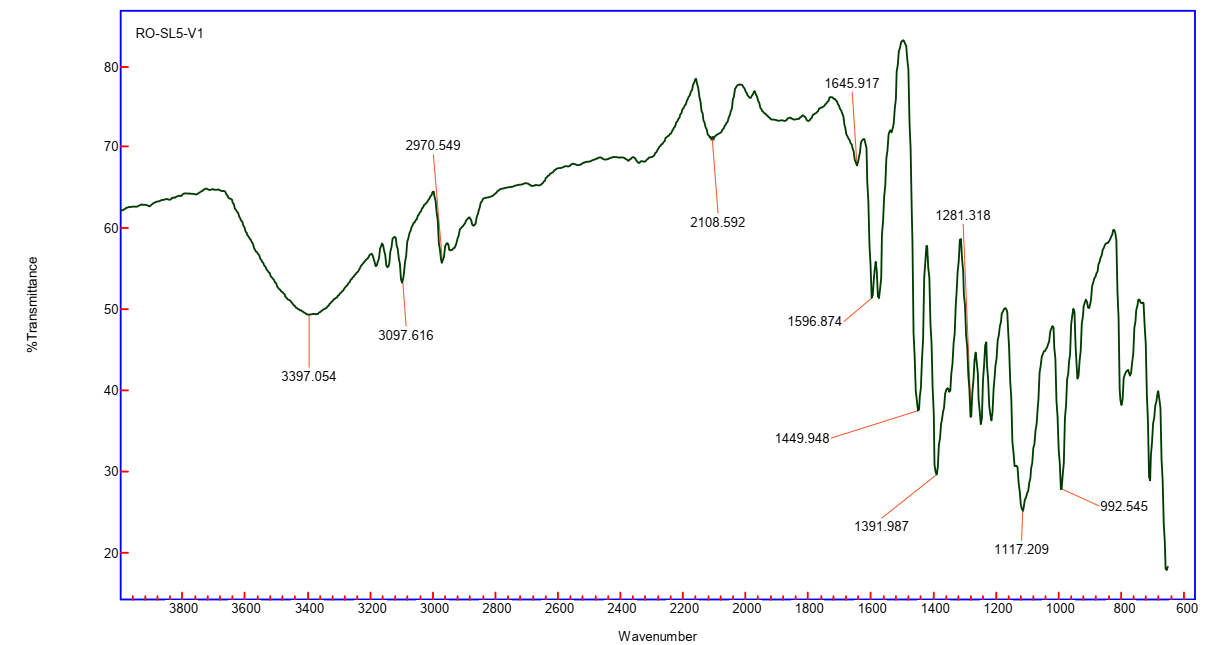 |
| --- |

**Fig. S20:** FT-IR spectrum of **4** with υ(C-H, aromatic) = 3397; υ(C-H, alkyl) = 3097; υ(C= N, imidazole) = 1645; υ(C=N, pyridine) = 1449; υ(C=S, imidazole) = 1117, **υ**(C-N, imidazole) = 992.

| 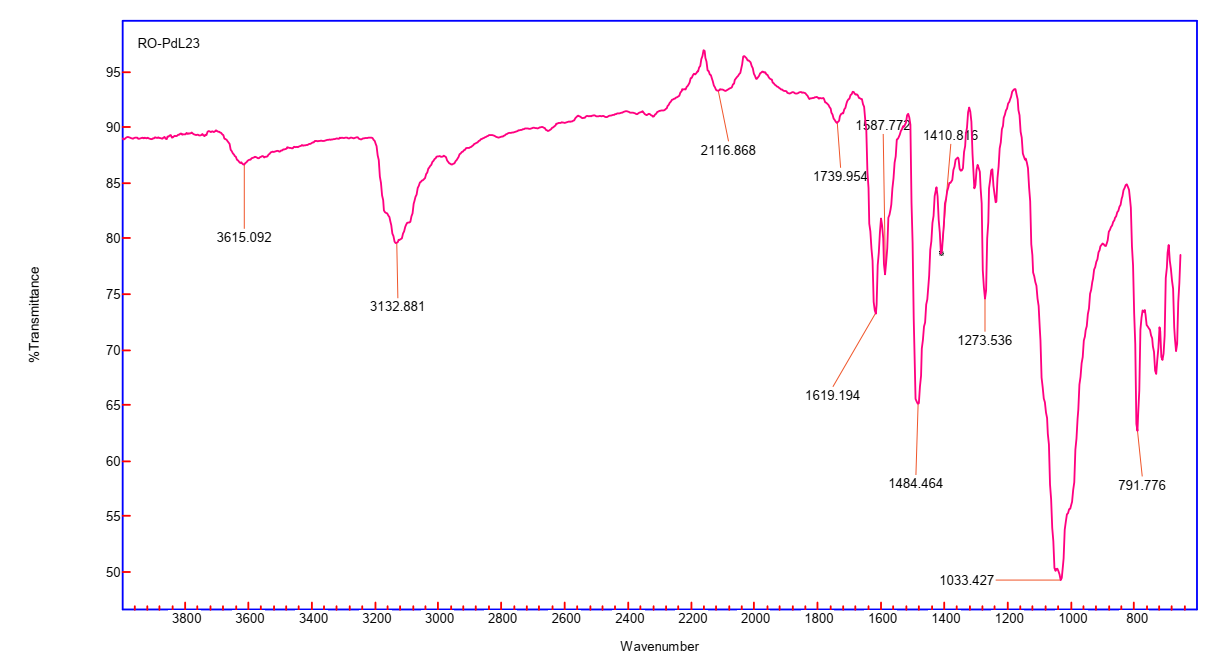 |
| --- |

## **Fig. S21:** FT-IR spectrum of **Pd1**, with υ(C-H, aromatic) = 3615; υ(C-H, alkyl) = 3132; υ(C= N, imidazole) = 1619; υ(C=N, pyridine) = 1587; υ(C-N, imidazole) = 1033. Shifts of the absorption bands at 1605 cm^-1^ (C=N, imidazole) and 1531 cm^-1^ (C=N, pyridine) in **1** to higher frequency values of 1619 and 1587 cm^-1^ respectively (**Pd1**), prove complexation.

| 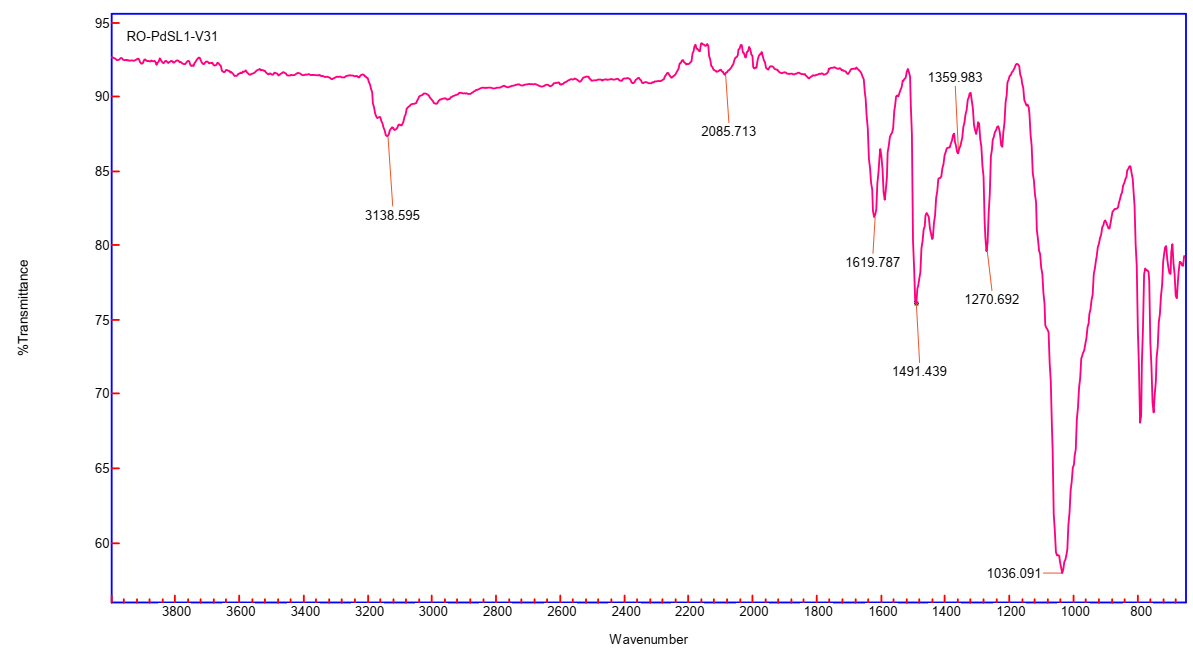 |
| --- |

## **Fig. S22:** FT-IR spectrum of **Pd2,** with υ(C-H, aromatic) = 3138; υ(C-H, alkyl) = 2989; υ(C= N, imidazole) = 1619; υ(C=N, pyridine) = 1491; υ(C-N, imidazole) = 1036. Shifts of the absorption bands at 1609 cm^-1^ (C=N, imidazole) and 1531 cm^-1^ (C=N, pyridine) in **2** to values of 1619 and 1491 cm^-1^ respectively in **Pd2** indicate complexation

##

| 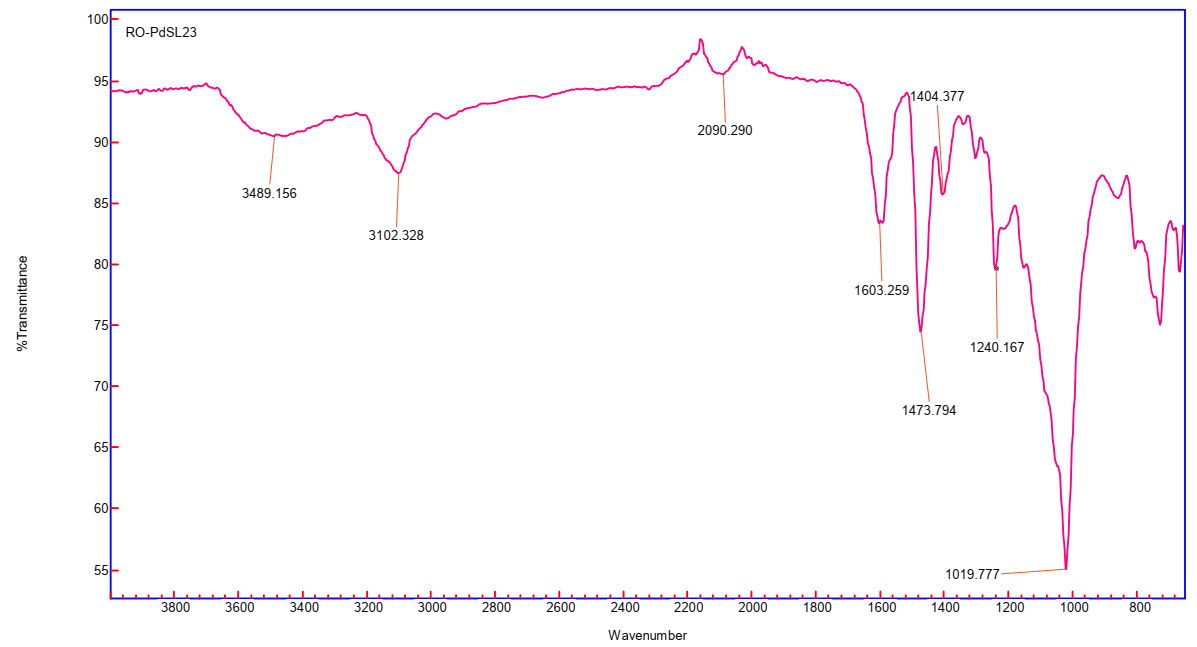 |
| --- |

## **Fig. S23:** FT-IR spectrum of **Pd3,** with υ(C-H, aromatic) = 3489; υ(C-H, alkyl) = 3102; υ(C= N, imidazole) = 1603; υ(C=N, pyridine) = 1473; υ(C=S, imidazole) = 1240, υ(C-N, imidazole) = 1019. Shifts of the absorption bands at 1575 cm^-1^ (C=N, pyridine) and 1142 (C=S, imidazole) in **3** to 1473 and 1240 cm^-1^ respectively (**Pd3**), confirm complexation.

| 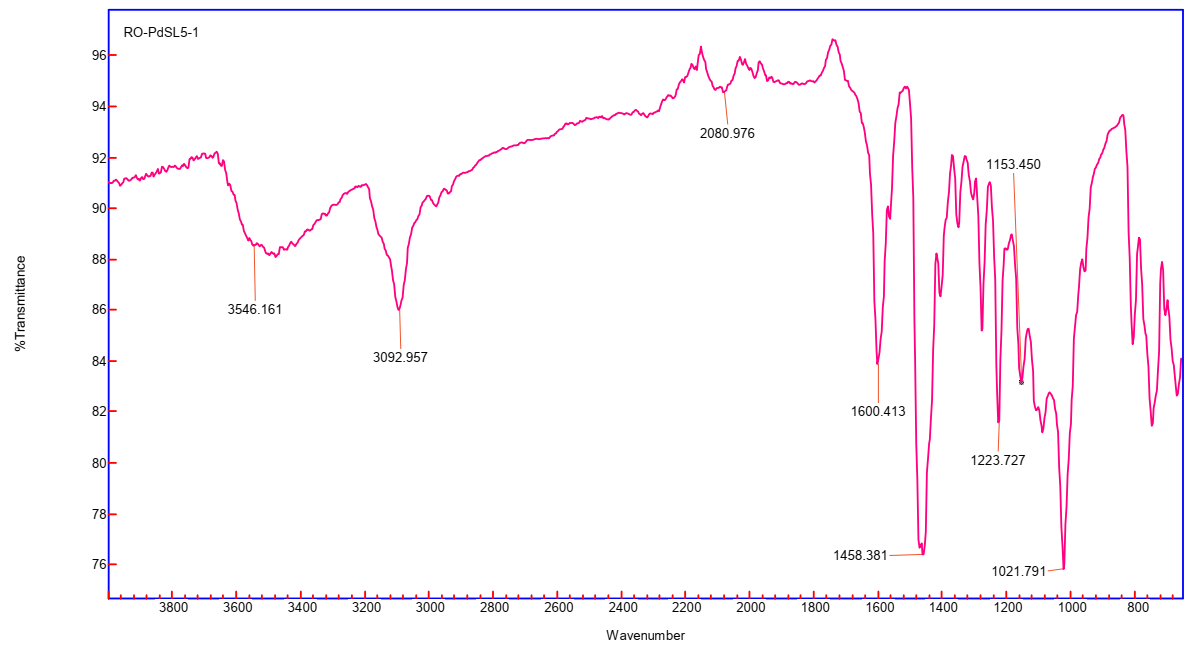 |
| --- |

## **Fig. S24:** FT-IR spectrum of **Pd4,** υ(C-H, aromatic) = 3546; υ(C-H, alkyl ) = 3092; υ(C= N, imidazole) = 1600; υ(C=N, pyridine) = 1458; υ(C=S) = 1153; υ(C-N, imidazole) = 1021. Shifts of the absorption bands at 1449 cm^-1^ (C=N, pyridine) and 1117 (C=S, imidazole) in **4** to 1600 and 1223 cm^-1^ respectively (**Pd4**), confirm complexation.

| 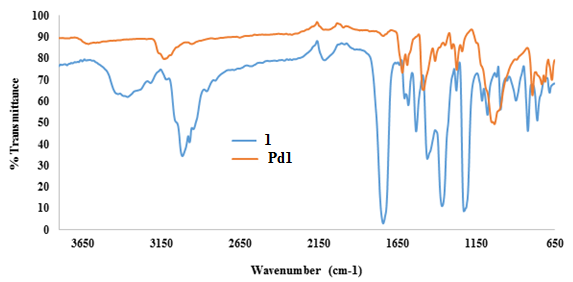 |
| --- |

## **Fig. S25**: Overlays of FT-IR spectra of **1** and the respective complex **Pd1**, indicating a shift of 1605 cm^-1^ υ (C=N, imidazole) and 1531 cm^-1^ υ (C=N, pyridine) (**1**) to 1619 and 1587 cm^-1^, respectively upon complexation **(Pd1)**.

# **13. LC-MS spectra**

| 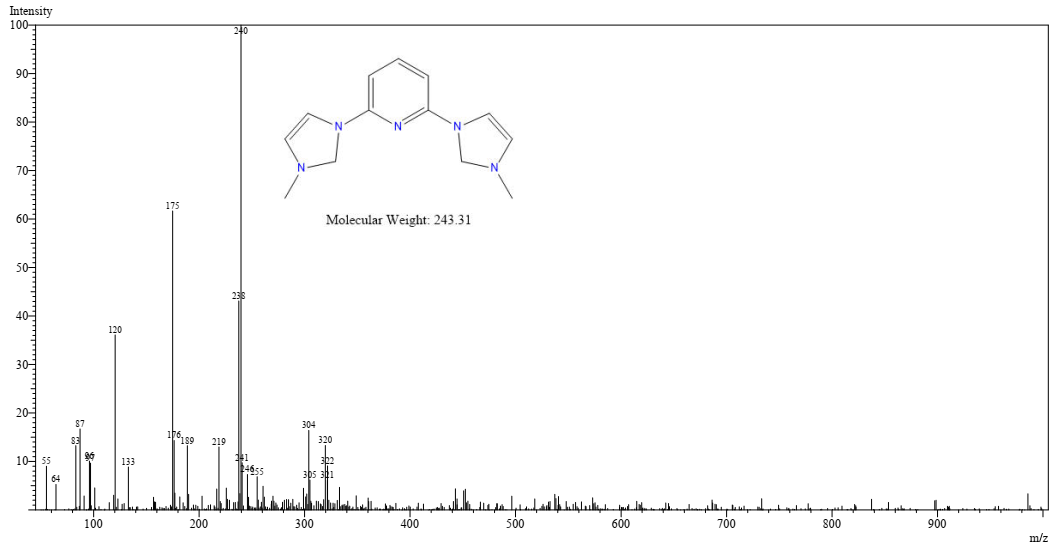 |
| --- |

## **Fig. S26:** Mass spectrum of **1** showing a parent peak m/z at 240 (100%), corresponding to the [M + Na] ^+^ adduct.

| 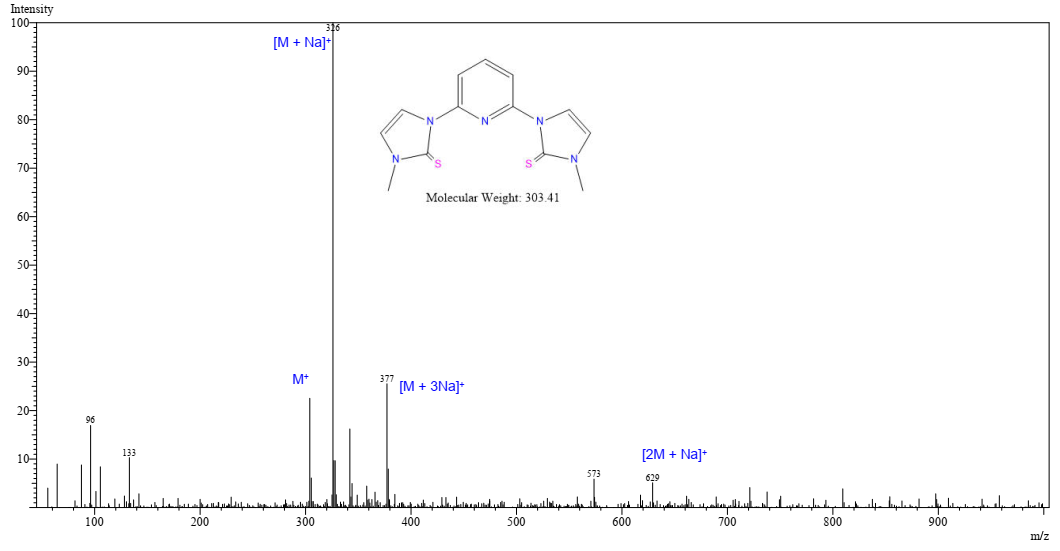 |
| --- |

## **Fig. S27:** Mass spectrum of **3**, showing a dominant peak m/z at 326 (100%), corresponding to the [M + Na] ^+^ adduct.

| 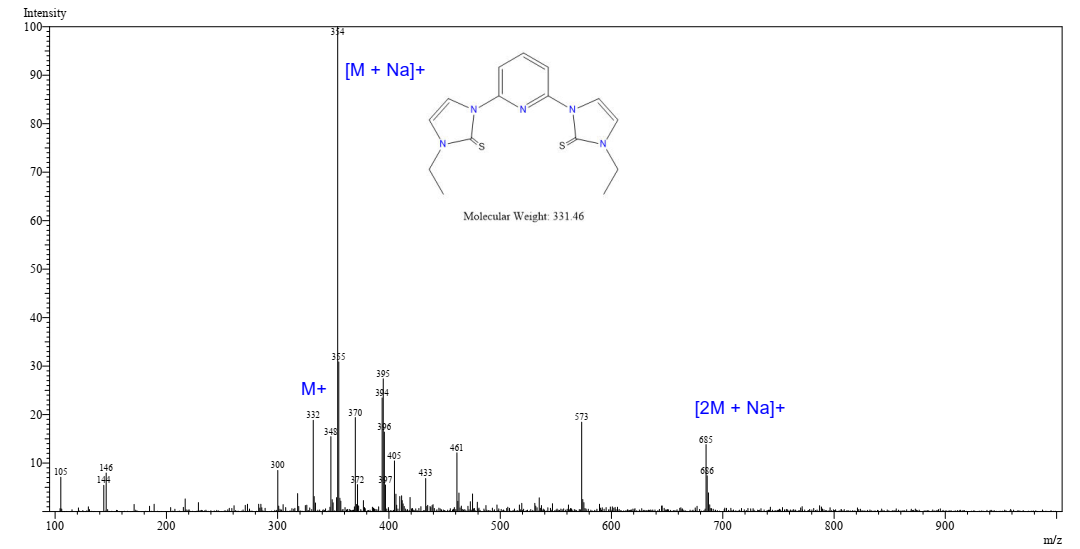 |
| --- |

## **Fig. S28:** Mass spectrum of **4** with *m/z* at 354 (100%) corresponding to the adduct [M+ Na)]^+^

| 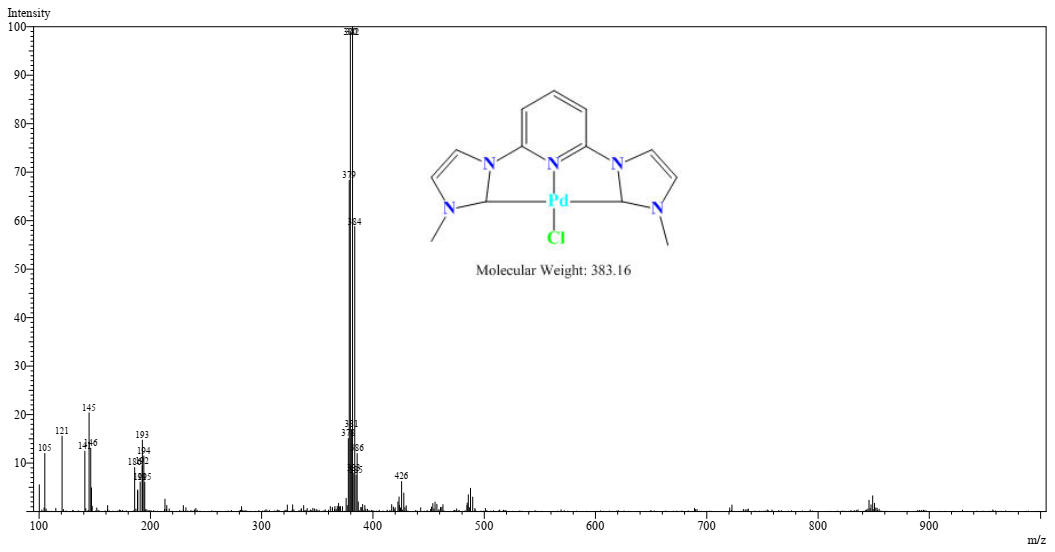 |
| --- |

## **Fig. S29a**: Mass spectrum of **Pd1** with *m/z* at 382 (100%) corresponding to the cationic specie [M-BF_4_)] ^+^

| 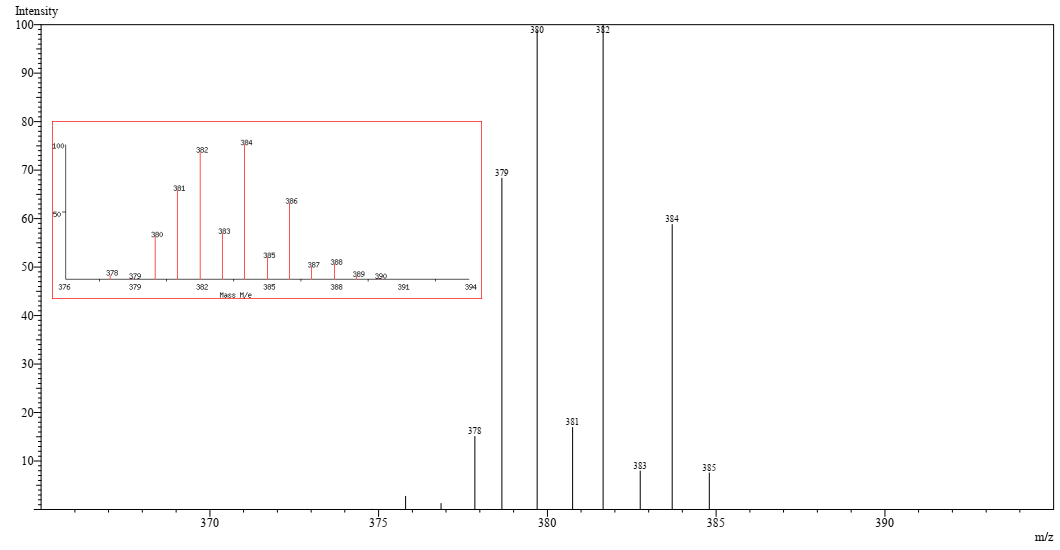 |
| --- |

## **Fig. S29b**: Expanded ESI mass spectrum of complex **Pd1** with *m/z* at 382 (100%) in agreement with the exact mass of 382.01. There was good agreement between the experimental and the calculated (inset) isotopic mass distribution patterns.

| 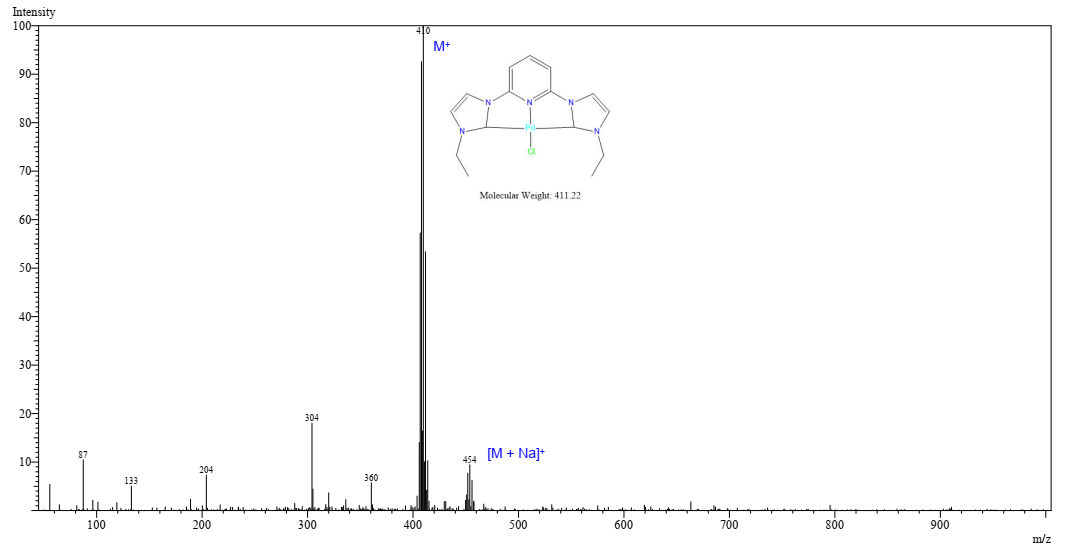 |
| --- |

## **Fig. S30:** Mass spectrum of **Pd2,** with *m/z* at 410 (100%) corresponding to the cationic specie [M-BF_4_)] ^+^


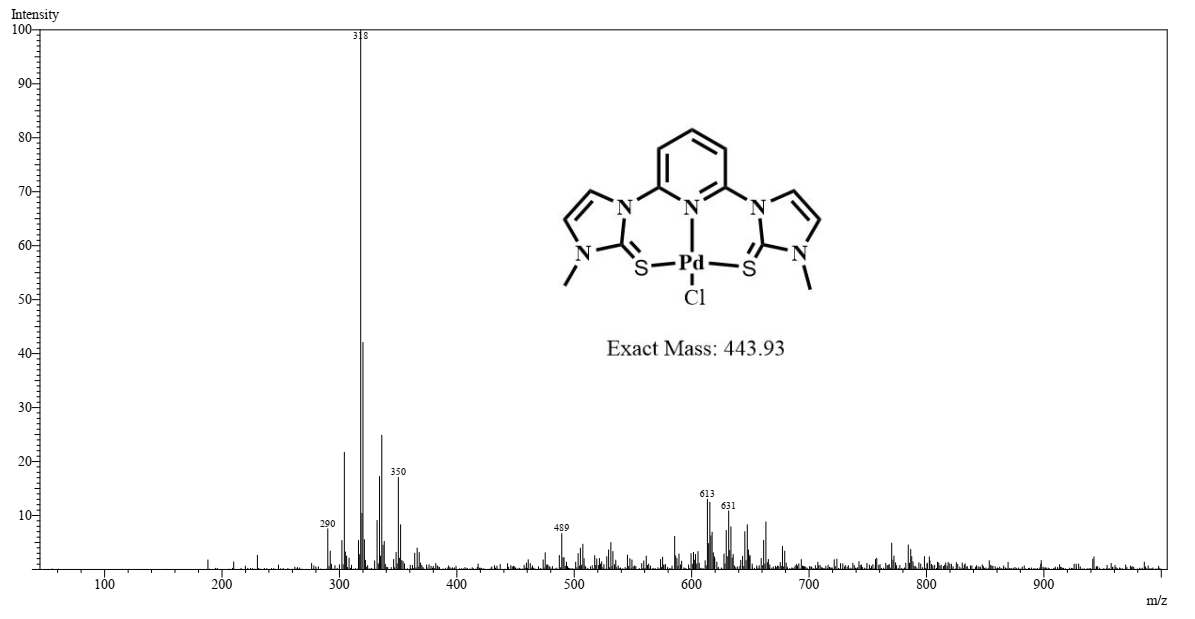


## **Fig. S31:** Mass spectrum of **Pd3** with *m/z* at 489 (8%) consistent with the species [(M-BF_4_) + 2Na] ^+^

| 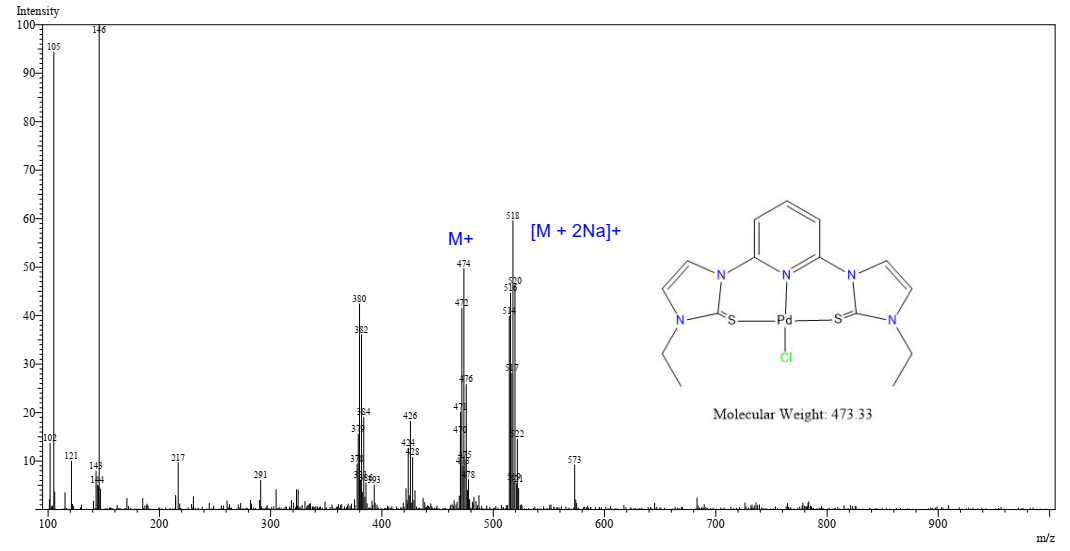 |
| --- |

## **Fig. S32:** Mass spectrum of **Pd4** with *m/z* at 474 (55%) corresponding to the cationic specie [M-BF_4_)] ^+^

# **14. DFT optimised structures**

| **Optimised molecular structure** | **HOMO map** | **LUMO map** | **Planarity** |
| --- | --- | --- | --- |
| 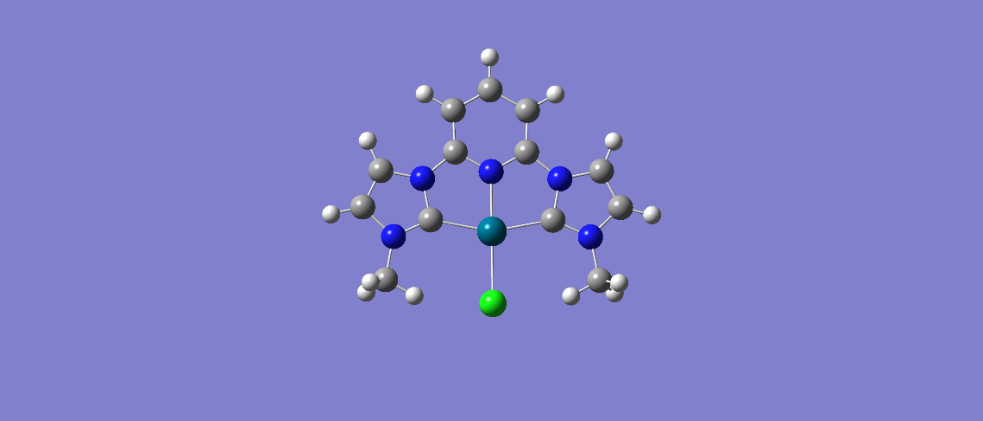  **Pd1** | 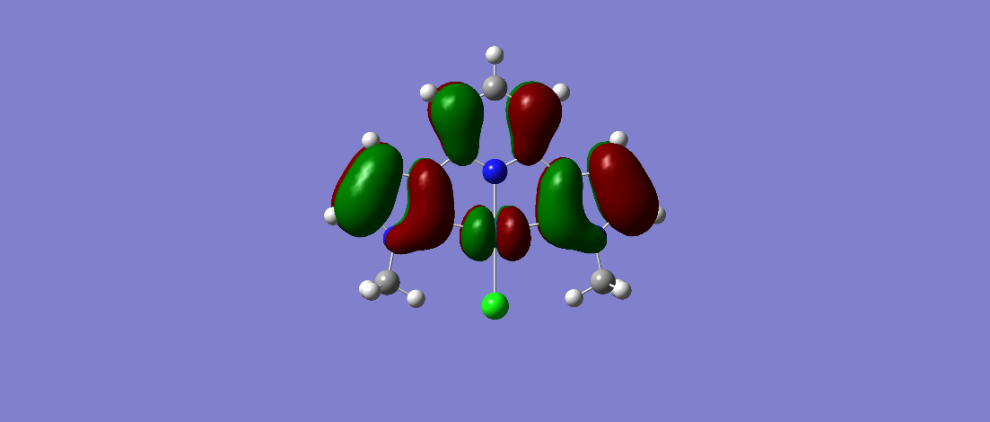 | 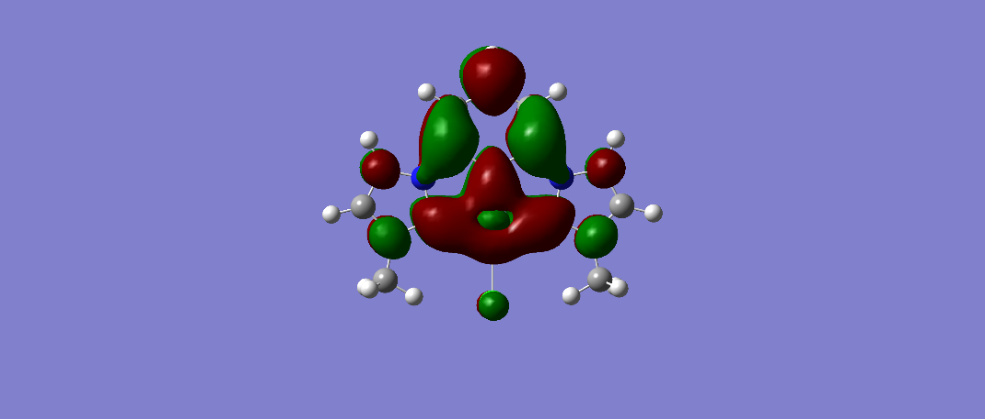 | 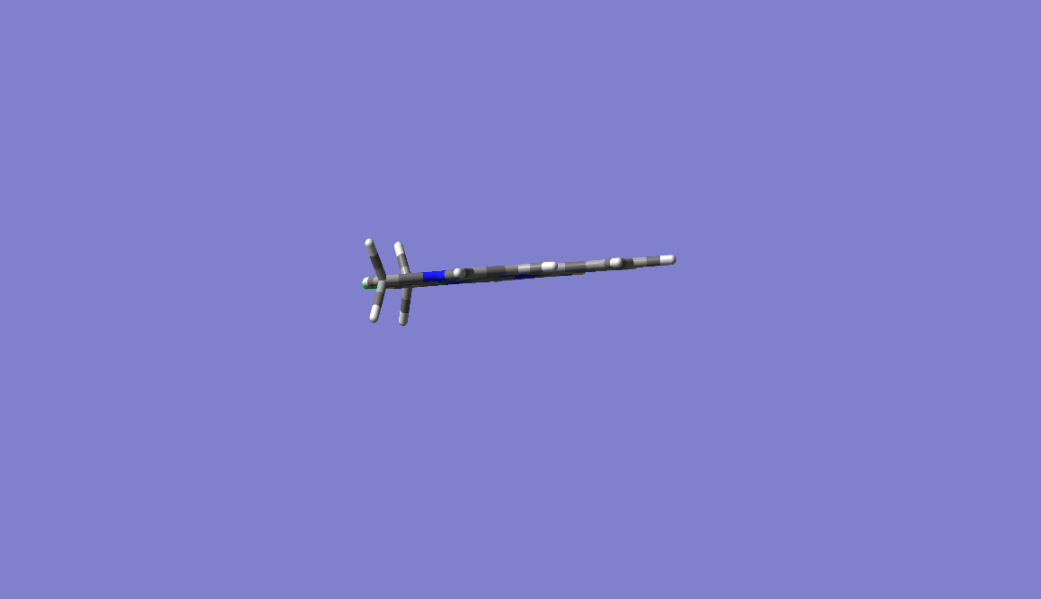 |
| 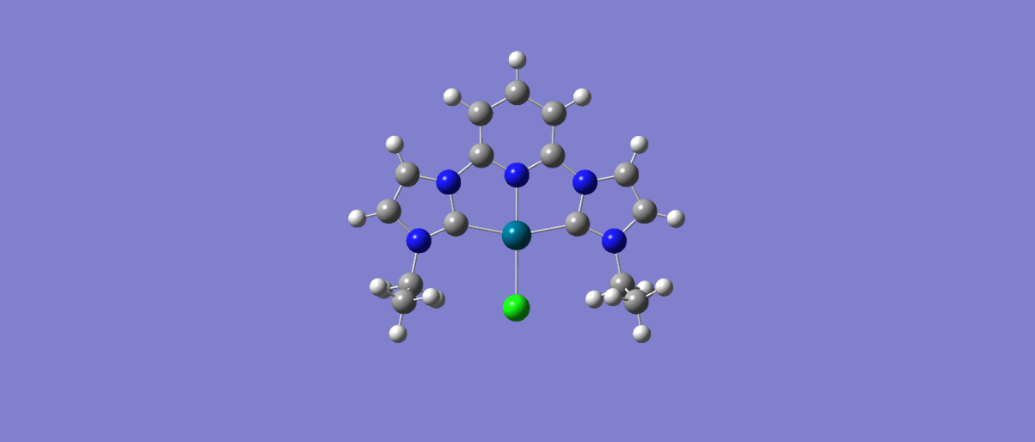  **Pd2** | 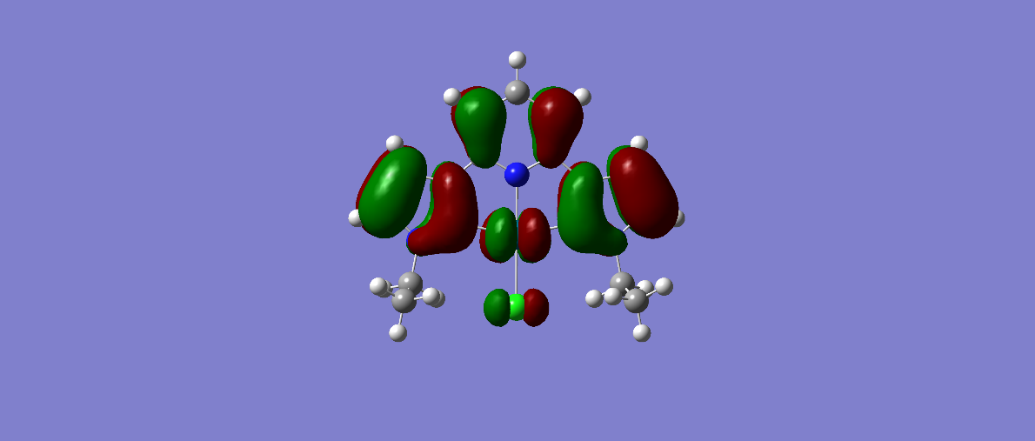 | 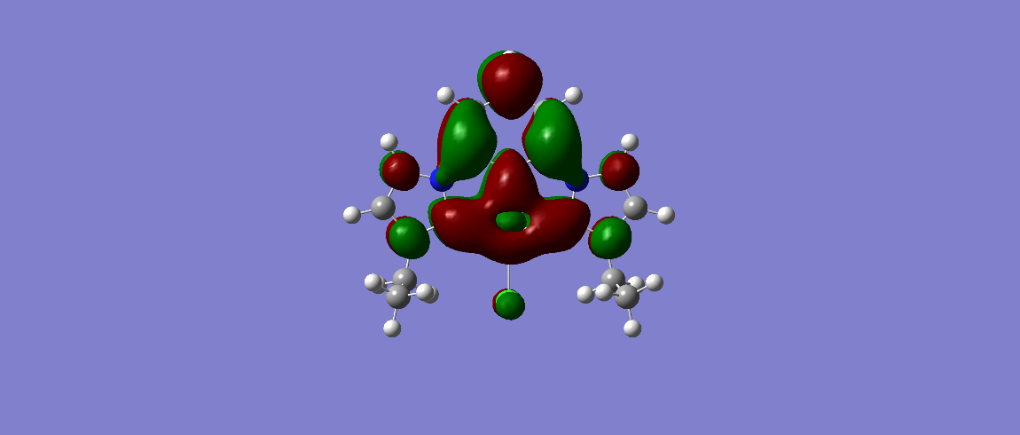 | 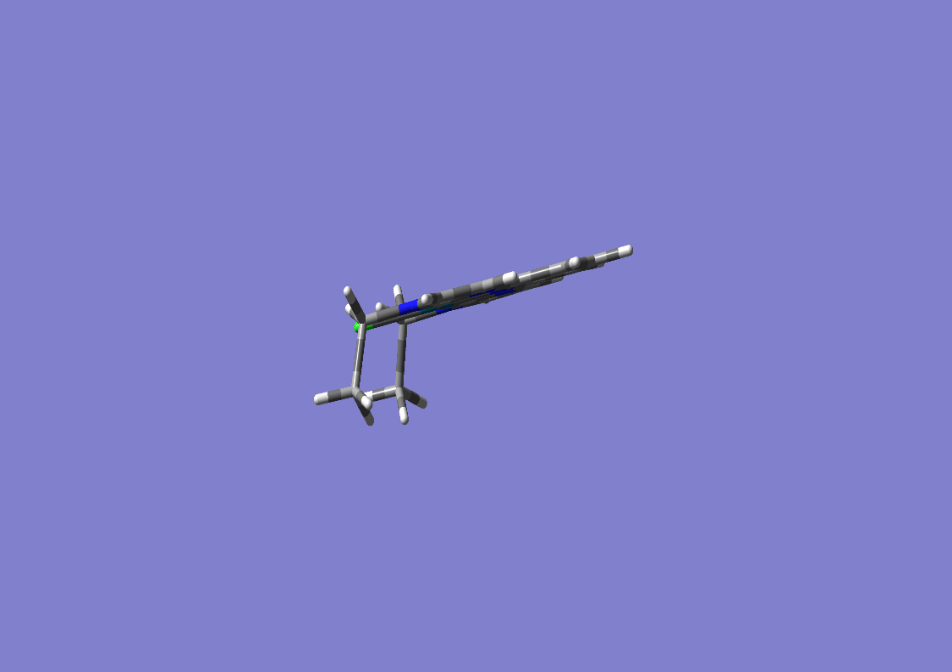 |
| 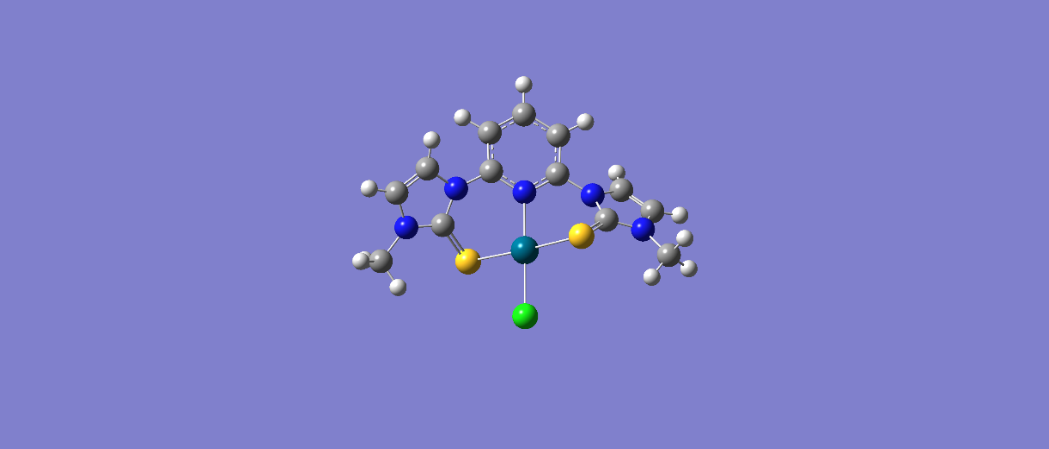  **Pd3** | 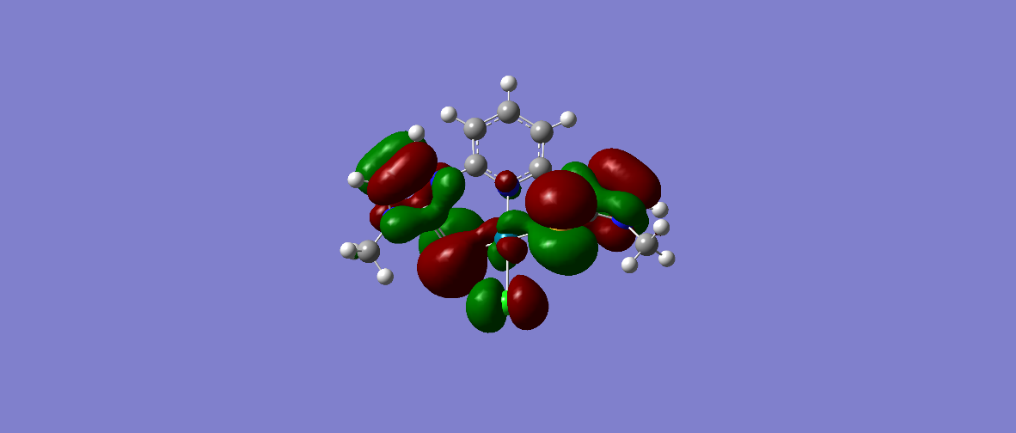 | 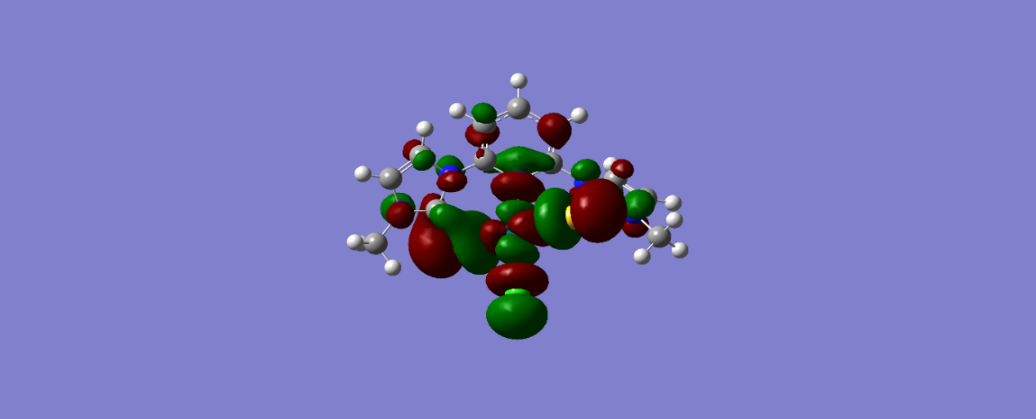 | 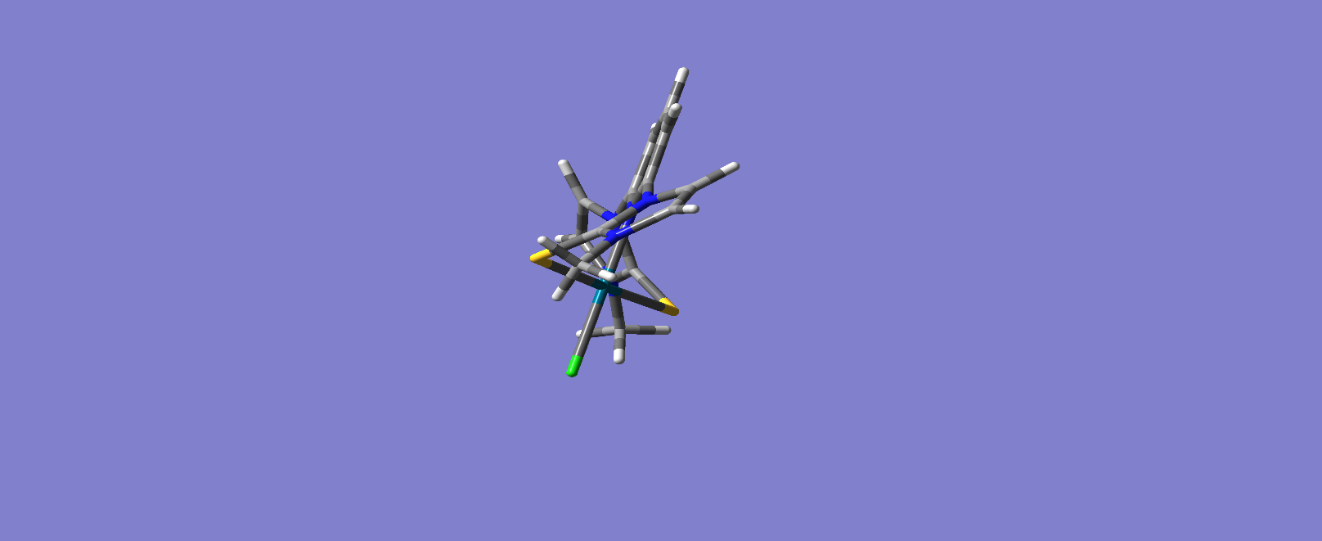 |
| 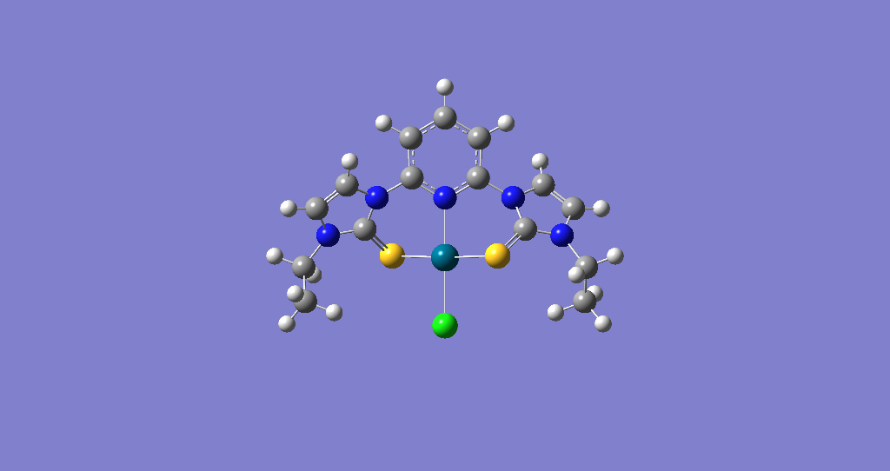  **Pd4** | 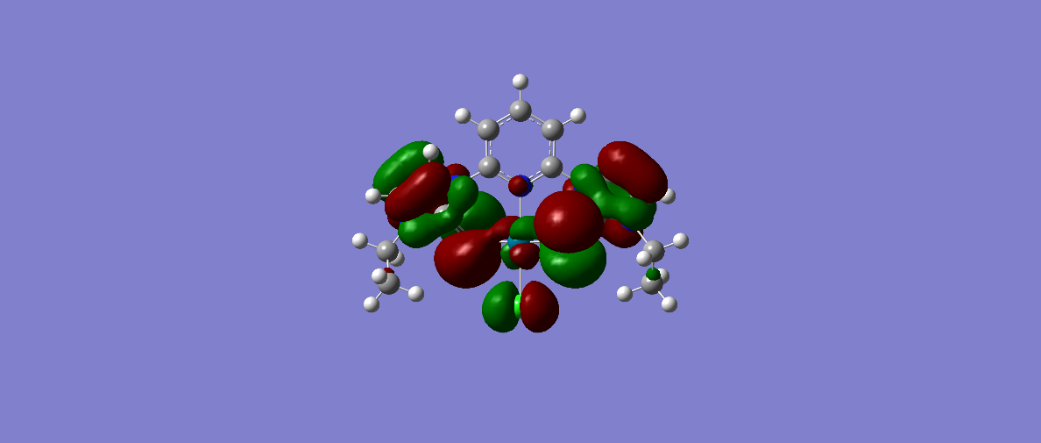 | 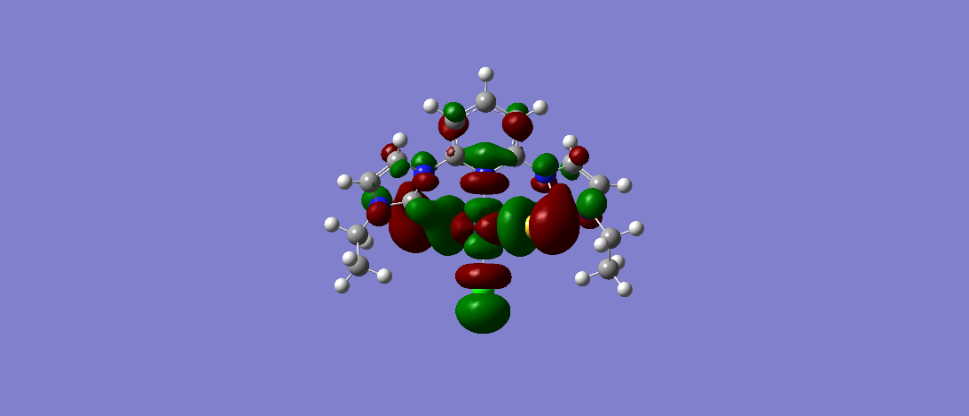 | 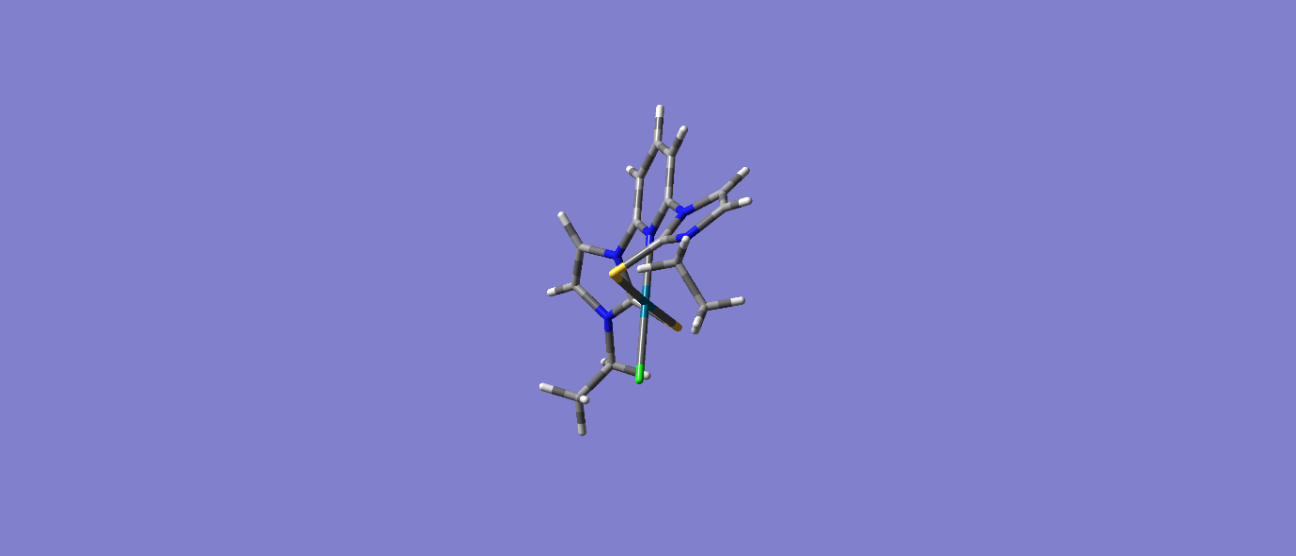 |

## **Fig. S33:** Frontier molecular orbitals (HOMO and LUMO), and planarity for complexes **Pd1**-**Pd4**

# **15. Solubility studies**

|   **(a)** |   **(b)** |
| --- | --- |
|   **(c)** |   **(d)** |

## **Fig. S34**: Typical UV-Vis absorption spectra of **Pd1** (a), **Pd2** (b), **Pd3** (c), **Pd4** (d), [Pd(II)] = 1.0 x 10^-5^ M, recorded as a function of time (from 0-72 h) in aqueous solution (Tris buffer at pH = 7.2), at room temperature.

# **16. Electrochemical studies**

|   **(a)** |   **(b)** |
| --- | --- |
|   **(c)** |   **(d)** |

## **Fig. S35:** Overlays of CV of **Pd1 (a)**, **Pd2 (b)**, **Pd3 (c)** and **Pd4 (d)** in DMSO solutions at 100 mV/s

# **17. Substitution kinetics studies**

|   **(a)** |   **(b)** |
| --- | --- |
|   **(c)** | |

## **Fig. S36**: Dependence of *k*_obs_ on the nucleophile concentration for chloride substitution of complexes (**a**) **Pd2-Pd4 (b) Pd3 (b) and (c) Pd4** at T = 298 K.

|   **(a)** |   **(b)** |
| --- | --- |
|   **(c)** | |

## **Fig. S37**: Eyring plots for the reactions of **complexes** (**a**) **Pd2-Pd4 (b) Pd3 (b) and (c) Pd4** with the nucleophiles in aqua, in 50 μM Tris-HCl buffer (pH = 7.2), and 50 mM NaCl and 10 mM NaCl and temperature range of 298-318 K.

# **18. DNA/BSA** **spectral studies**

|   **(a)** |   **(b)** |
| --- | --- |
|   **(c)** | |

## **Fig. S38:** Absorption spectra of (**a**) **Pd2** [20 µM], **(b) Pd3** [20 µM], **(c) Pd4** [20 µM] in Tris-HCl/50 mM buffer at pH 7.2 upon addition of CT-DNA (0- 80 μM). The arrow shows the change in absorbance upon increasing the CT-DNA concentration. Inset: plot of [CT-DNA] *vs* [DNA]/(ε_a_- ε_f_).

|  |  |
| --- | --- |
|  |  |

## **Fig. S39:** (**a**) Fluorescence emission spectra of EB bounded to CT-DNA in the presence of **Pd2**: [EB] = 20 μM, [CT-DNA] = 20 μM and [**Pd2**] = 0-200 μM. The arrow shows the intensity changes upon increasing the **Pd2** complex concentration. **(b)** Stern-Volmer plot of *I*_o_/*I* *vs* [Q]. **(c)** Scatchard plot of log[(*I*_o_–I)/*I*] *vs* log[Q].

|  |  |
| --- | --- |
|  |  |

## **Fig. S40**: **(a)** Fluorescence emission spectra of EB bounded to CT-DNA in the presence of **Pd3**: [EB] = 20 μM, [CT-DNA] = 20 μM and **[Pd3]** = 0-200 μM. The arrow shows the intensity changes upon increasing the Pd3 complex concentration. (**b**) Stern-Volmer plot of *I*_o_/*I* *vs* [Q]. **(c)** Scatchard plot of log[(*I*_o_–I)/*I*] *vs* log[Q].

|  |  |
| --- | --- |
|  |  |

## **Fig. S41**: **(a)** Fluorescence emission spectra of EB bounded to CT-DNA in the presence of **Pd4**: [EB] = 20 μM, [CTDNA] = 20 μM and [**Pd4**] = 0-200 μM. The arrow shows the intensity changes upon increasing the **Pd4** complex concentration. **(b)**; Stern-Volmer plot of *I*_o_/*I* *vs* [Q]. **(c)** Scatchard plot of log[(*I*_o_–I)/*I*] *vs* log[Q].

|  |  |
| --- | --- |
|  |  |

## **Fig. S42: (a):** Fluorescence emission spectra of BSA in the absence and presence of **Pd2**: [BSA] = 12.0 μM and [**Pd2**] = 0-200 μM. The arrow shows the intensity changes upon increasing the **Pd2** concentration. **(b)**: Stern-Volmer plot of *I*_o_/*I* *vs* [Q] and **(c)**: Scatchard plot of log[(*I*_o_–I)/*I*] *vs* log[Q].

|  |  |
| --- | --- |
|  |  |

## **Fig. S43: (a)** Fluorescence emission spectra of BSA in the absence and presence of **Pd3**: [BSA] = 1.2 μM and [**Pd3**] = 0 -25 μM. The arrow shows the intensity changes upon increasing the **Pd3** concentration. **(b)**: Stern-Volmer plot of *I*_o_/*I* *vs* [Q] and **(c)**: Scatchard plot of log[(*I*_o_–I)/*I*] *vs* log[Q].

|  |  |
| --- | --- |
|  |  |

## **Fig. S44: (a)** Fluorescence emission spectra of BSA in the absence and presence of **Pd4**: [BSA] = 1.2 μM and [**Pd4**] = 0-25 μM. The arrow shows the intensity changes upon increasing the **Pd4** concentration. **(b)**: Stern-Volmer plot of *I*_o_/*I* *vs* [Q] and **(c):** Scatchard plot of log[(*I*_o_–I)/*I*] *vs* log[Q].

# **19. *In silico* approach**

| 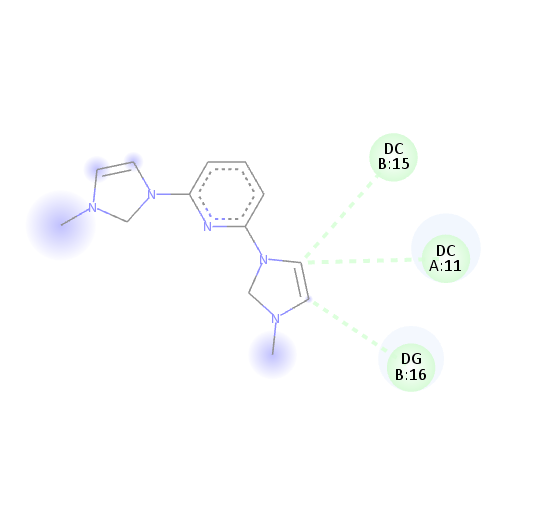  **(a)** | 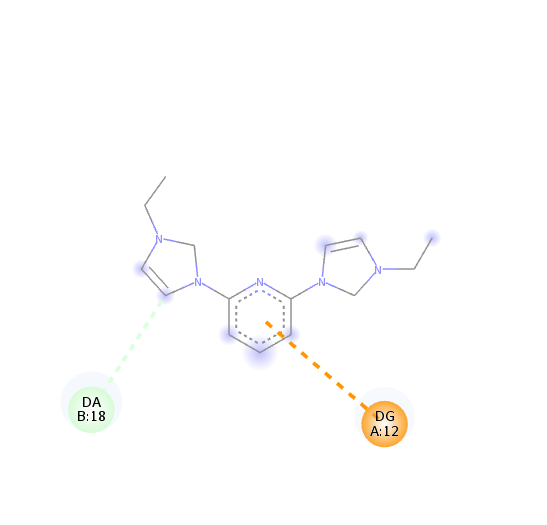  **(b)** |
| --- | --- |
| 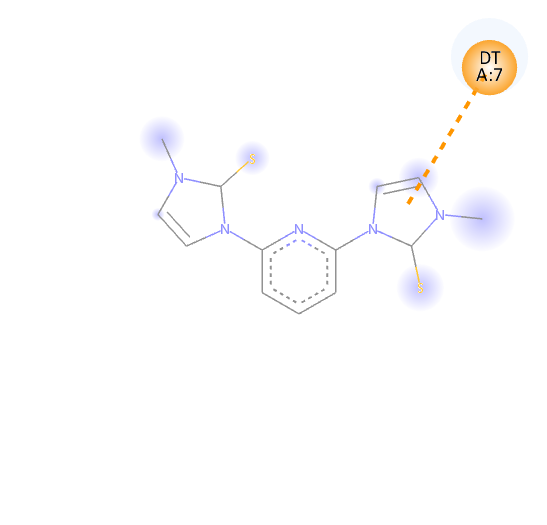  **(c)** | 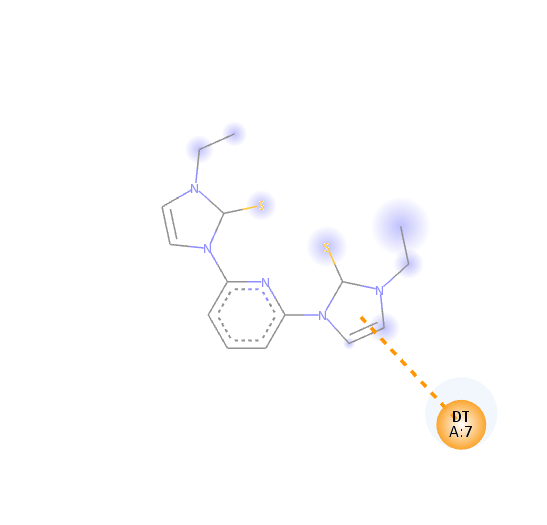  **(d)** |

## **Fig. S45:** 2D Interaction diagrams of **Pd1** (**a**), **Pd2** (**b**), **Pd3** (**c**), **Pd4** (**d**) with DNA.

# **20. X-ray structure**

## **Table S1:** Crystal data and structure refinement for complex **Pd2**

| **Parameters** | **Pd2** |
| --- | --- |
| Empirical formula | C_15_H_17_BClF_4_N_5_Pd |
| Formula weight | 495.99 |
| Temperature | 100(2) K |
| Wavelength | 0.71073 Å |
| Crystal system | Monoclinic |
| Space group | P 21/n |
| Unit cell dimensions |  |
|  | a = 7.5773(4) Å  b = 24.1654(11) Å  c = 10.3250(5) Å  α = 90°; β =110.791(2)°; γ = 90° |
| Volume | 1767.48(15) Å3 |
| Z | 4 |
| Density (calculated) | 1.864 Mg/m3 |
| Absorption coefficient | 1.252 mm-1 |
| F(000) | 984 |
| Crystal size | 0.222 x 0.091 x 0.058 mm3 |
| Index ranges | -9<=h<=9, -30<=k<=30, -13<=l<=13 |
| Reflections collected | 27251 |
| Independent reflections | 3681 [R(int) = 0.0320] |
| Completeness to theta = 25.242° | 98.4 % |
| Refinement method | Full-matrix least-squares on F2 |

## **Table S2**:  Summary of selected DFT-calculated data for complexes, **Pd1-PdL_4_**

| **Properties** | **PdL_1_** | **PdL_2_** | **PdL_3_** | **PdL_4_** |
| --- | --- | --- | --- | --- |
| **NBO charge** |  |  |  |  |
| Pd^2+^ | 0.276 | 0.262 | -0.053 | -0.061 |
| *Cis*-C | 0.164 | 0.171 | - | - |
| *Cis*-S | - | - | 0.126 | 0.124 |
| **Bond lengths (Å)** |  |  |  |  |
| Pd-N | 2.007 | 2.007 | 2.111 | 2.110 |
| Pd- C | 2.065 | 2.063 | - | - |
| Pd-S | - | - | 2.447 | 2.450 |
| Pd-Cl | 2.414 | 2.410 | 2.400 | 2.399 |
| **Bond angles (º)** |  |  |  |  |
| C – Pd – C | 158.504 | 158.563 | - |  |
| S – Pd – S | - | - | 177.619 | 177.132 |
| N – Pd – C | 79.252 | 79.281 | - |  |
| N – Pd – S | - | - | 88.812 | 88.565 |
| **MO energy (eV)** |  |  |  |  |
| -E_HOMO_ | 7.0712 | 6.9462 | 6.6734 | 6.6007 |
| -E_LUMO_ | 2.7582 | 2.7492 | 3.3323 | 3.3427 |
| ΔE_LUMO–HOMO_ | 4.3130 | 4.2970 | 3.3341 | 3.2580 |
| Electrophilicity index (*ω*) | 5.6003 | 5.4689 | 7.5067 | 7.5868 |
| **Dipole moment (*D*)** | 12.3536 | 12.9170 | 18.6181 | 18.6725 |

# **21. Kinetic studies tables**

## **Table S3**: Average values of *k*_obs_ (s^-1^) for the substitution of chloride from **Pd1** by the nucleophile at T = 298 K in aqua media, 50 μM Tris-HCl buffer (pH = 7.2), and 50 mM NaCl.

| **[Nu]** | ***k*_obs, S_^-1^** | | |
| --- | --- | --- | --- |
|  | **Tu** | **L-Met** | **5’-GMP** |
| 0.004 | 11.112 | 1.01 | 0.238 |
| 0.008 | 21.92 | 2.259 | 0.452 |
| 0.012 | 32.72 | 3.355 | 0.705 |
| 0.016 | 44.64 | 4.199 | 0.975 |
| 0.02 | 56.5 | 5.107 | 1.117 |

## **Table S4**: Temperature dependence of *k*_2_ M^-1^s^-1^ for the reactions of **Pd1** by the nucleophiles in aqueous medium, 50 μM Tris-HCl buffer (pH = 7.2), and 50 mM NaCl and temperature range of 298-318 K.

| **1/T, K^-1^** | In(*k*_2_/T) | | |
| --- | --- | --- | --- |
|  | **Tu** | **L-Met** | **5-GMP** |
| 0.00336 | 2.21374 | -0.06379 | -1.6238 |
| 0.00325 | 2.3949 | 0.15041 | -1.31149 |
| 0.00314 | 2.5661 | 0.39051 | -1.0109 |
| 0.00305 | 2.7349 | 0.60291 | -0.65799 |

## **Table S5**: Average values of *k*_obs_ (s^-1^) for the substitution of chloride from Pd2 by the nucleophile at T = 298 K in aqua media, 50 μM Tris-HCl buffer (pH = 7.2), and 50 mM NaCl.

| **[Nu]** | ***k*_obs, S_^-1^** | | |
| --- | --- | --- | --- |
|  | **Tu** | **L-Met** | **5’-GMP** |
| 0.004 | 8.421 | 0.808 | 0.192 |
| 0.008 | 17.042 | 1.696 | 0.339 |
| 0.012 | 25.164 | 2.494 | 0.524 |
| 0.016 | 33.489 | 3.0712 | 0.782 |
| 0.02 | 43.002 | 4.1879 | 0.801 |

## **Table S6**: Temperature dependence of *k*_2_ M^-1^s^-1^ for the reactions of **Pd2** by the nucleophiles in aqueous medium, in 50 μM Tris-HCl buffer (pH = 7.2), and 50 mM NaCl and temperature range of 298-318 K.

| **1/T, K^-1^** | In(*k*_2_/T) | | |
| --- | --- | --- | --- |
|  | **Tu** | **L-Met** | **5-GMP** |
| 0.00336 | 1.95117 | -0.36036 | -1.92051 |
| 0.00325 | 2.13268 | -0.10902 | -1.57949 |
| 0.00314 | 2.32791 | 0.15112 | -1.26728 |
| 0.00305 | 2.49871 | 0.39245 | -0.94597 |

## **Table S7**: Average values of *k*_obs_ (s^-1^) for the substitution of chloride from **Pd3** by the nucleophile at T = 298 K in aqua media, 50 μM Tris-HCl buffer (pH = 7.2), and 50 mM NaCl.

| **[Nu]** | ***k*_obs, S_^-1^** | | |
| --- | --- | --- | --- |
|  | **Tu** | **L-Met** | **5’-GMP** |
| 0.004 | 5.052 | 0.568 | 0.108 |
| 0.008 | 10.804 | 1.236 | 0.296 |
| 0.012 | 15.456 | 1.604 | 0.314 |
| 0.016 | 21.008 | 2.272 | 0.452 |
| 0.02 | 26.461 | 2.749 | 0.498 |

## **Table S8**: Temperature dependence of *k*_2_ M^-1^s^-1^ for the reactions of **Pd3** by the nucleophiles in aqueous medium, in 50 μM Tris-HCl buffer (pH = 7.2), and 50 mM NaCl and temperature range of 298-318 K.

| **1/T, K^-1^** | In(*k*_2_/T) | | |
| --- | --- | --- | --- |
|  | **Tu** | **L-Met** | **5’-GMP** |
| 0.00336 | 1.46375 | -0.80174 | -2.43261 |
| 0.00325 | 1.86527 | -0.32132 | -1.94139 |
| 0.00314 | 2.24078 | 0.19288 | -1.39028 |
| 0.00305 | 2.50125 | 0.71622 | -0.79905 |

## **Table S9**: Average values of *k*_obs_ (s^-1^) for the substitution of chloride from **Pd4** by the nucleophile at T = 298 K in aqua media, 50 μM Tris-HCl buffer (pH = 7.2), and 50 mM NaCl.

| **[Nu]** | ***k*_obs, S_^-1^** | | |
| --- | --- | --- | --- |
|  | **Tu** | **L-Met** | **5’-GMP** |
| 0.004 | 3.8891 | 0.4351 | 0.0854 |
| 0.008 | 7.7698 | 0.8525 | 0.1712 |
| 0.012 | 11.4772 | 1.2983 | 0.2451 |
| 0.016 | 15.185 | 1.6521 | 0.3931 |
| 0.02 | 18.7852 | 2.1881 | 0.41521 |

## **Table S10**: Temperature dependence of *k*_2_ M^-1^s^-1^ for the reactions of **Pd4** by the nucleophiles in aqueous medium 50 μM Tris-HCl buffer (pH = 7.2), and 50 mM NaCl and temperature range of 298-318 K.

| **1/T, K^-1^** | In(*k*_2_/T) | | |
| --- | --- | --- | --- |
|  | **Tu** | **L-Met** | **5’-GMP** |
| 0.00336 | 1.16612 | -1.01319 | -2.68025 |
| 0.00325 | 1.58394 | -0.52098 | -2.16011 |
| 0.00314 | 2.0054 | -0.00845 | -1.60801 |
| 0.00305 | 2.24665 | 0.50275 | -1.00731 |

# **22. Molecular docking investigations**

## **Table S11:** Binding affinities of Pd-metal complexes with DNA and BSA

| **Complex** | **DNA binding affinity** **(kcal/mol)** | **BSA binding affinity (kcal/mol)** |
| --- | --- | --- |
| **Pd1** | -6.7 | -6.9 |
| **Pd2** | -6.6 | -6.5 |
| **Pd3** | -6.4 | -6.7 |
| **Pd4** | -6.4 | -6.6 |

The more negative binding free energy, the stronger the binding affinity between DNA/BSA and Pd-complexes. Generally, the lower relative binding energies of the complexes could be ascribed to their non-planar nature (as shown in DFT computations, **Fig. S32**).

**Table S12**: Intermolecular interactions of Pd-complexes with DNA nucleotides and BSA amino acids

| **Complex** | **DNA nucleotides** | **Protein amino acids** |
| --- | --- | --- |
| **Pd1** | C-H bond (DC11 & 15 and DG 16) | C-H bond (ARG144) and π-cation (ARG458) |
| **Pd2** | π-anion (DG12) and C-H bond (DA18) | π- π T-shaped (TYR400, ALA405, and LEU528) and π-alkyl (MET547) |
| **Pd3** | π-anion (DT 7) | Conventional H bond (LEU189), C-H bond (GLU186), π-sulfur (HIS145), π -alkyl (LYS431, ARG458 and ILE455) and π - σ (ALA193) |
| **Pd4** | π-anion (DT7) | Conventional H bond (LEU189), C-H bond (GLU186), π-sulfur (HIS145), π -alkyl (LYS431, ARG458 and ILE455) and π - σ (ALA193), amide π-stacked |

# **23. References**

APeX B (2010) SAINT and SADABS. Bruker AXS Inc, Madison, Wisconsin, USA

Sheldrick GM (2015) Crystal structure refinement with SHELXL. Acta Cryst C 71: 3-8

Farrugia LJ (2012) WinGX and ORTEP for Windows: an update. J Appl Crystallogr 45: 849-54

Frisch M, Trucks G, Schlegel H, Scuseria G, Robb M, Cheeseman J, et al. (2010) Petersson et. al. Gaussian. Inc, Wallingford CT

Cossi M, Rega N, Scalmani G, Barone V (2003) Energies, structures, and electronic properties of molecules in solution with the C‐PCM solvation model. J Comput Chem 24: 669-81

Tobe ML, Burgess J. Inorganic reaction mechanisms. Longman; 1999.

Atwood JD. Inorganic and organometallic reaction mechanisms. VCH Publishers; 1997.

Eyring H (1935) The activated complex in chemical reactions. J Chem Phys 3: 107-15

Gray HB, Olcott RJ (1962) Kinetics of the Reactions of Diethylenetriamineaquoplatinum (II) Ion with Different Ligands. Inorg Chem 1: 481-5

Omondi RO, Bellam R, Ojwach SO, Jaganyi D, Fatokun AA (2020a) Palladium (II) complexes of tridentate bis (benzazole) ligands: Structural, substitution kinetics, DNA interactions and cytotoxicity studies. J Inorg Biochem 111156

Omondi RO, Sibuyi NR, Fadaka AO, Meyer M, Jaganyi D, Ojwach SO (2021) Role of π-conjugation on the coordination behaviour, substitution kinetics, DNA/BSA interactions, and in vitro cytotoxicity of carboxamide palladium (ii) complexes. Dalton Trans 50: 8127-43

Omondi RO, Bellam R, Ojwach SO, Jaganyi D, Fatokun AA (2020b) Palladium (II) complexes of tridentate bis (benzazole) ligands: Structural, substitution kinetics, DNA interactions and cytotoxicity studies. J Inorg Biochem 210: 111156

Parker C, Rees W (1962) Fluorescence spectrometry. A review. Analyst 87: 83-111

Trott O, Olson AJ (2010) AutoDock Vina: improving the speed and accuracy of docking with a new scoring function, efficient optimization, and multithreading. J Comput Chem 31: 455-61
